# Lateral fenestrations in the extracellular domain of the glycine receptor contribute to the main chloride permeation pathway

**DOI:** 10.1126/sciadv.adc9340

**Published:** 2022-10-14

**Authors:** Adrien H. Cerdan, Laurie Peverini, Jean-Pierre Changeux, Pierre-Jean Corringer, Marco Cecchini

**Affiliations:** ^1^ Institut de Chimie de Strasbourg, UMR7177, CNRS, Université de Strasbourg, F-67083 Strasbourg Cedex, France.; ^2^ Institut Pasteur, Université Paris Cité, CNRS UMR 3571, Channel-Receptors Unit, Paris, France.; ^3^ Kavli Institute for Brain and Mind, University of California, San Diego, La Jolla, CA, USA.; ^4^ Collège de France, Paris, France.

## Abstract

Glycine receptors (GlyRs) are ligand-gated ion channels mediating signal transduction at chemical synapses. Since the early patch-clamp electrophysiology studies, the details of the ion permeation mechanism have remained elusive. Here, we combine molecular dynamics simulations of a zebrafish GlyR-α1 model devoid of the intracellular domain with mutagenesis and single-channel electrophysiology of the full-length human GlyR-α1. We show that lateral fenestrations between subunits in the extracellular domain provide the main translocation pathway for chloride ions to enter/exit a central water-filled vestibule at the entrance of the transmembrane channel. In addition, we provide evidence that these fenestrations are at the origin of current rectification in known anomalous mutants and design de novo two inward-rectifying channels by introducing mutations within them. These results demonstrate the central role of lateral fenestrations on synaptic neurotransmission.

## Teaser

Extracellular chloride ions access the glycine receptor pore via lateral fenestrations outdating the standard apical model.

## INTRODUCTION

Glycine receptors (GlyRs) contribute to fast synaptic inhibition and mediate muscle tone regulation, motor coordination, processing of vision and audition, and pain sensation (*1*, *2*). Several inherited mutations of GlyRs are linked to autism, temporal lobe epilepsy, and hyperekplexia (startle disease) (*3*). GlyRs are pentameric ligand-gated ion channels (pLGICs) that are found as homopentamers of α subunits (α1 to α4) or heteropentamers combining α and β subunits. The α1 GlyR is a symmetrical homopentamer, each subunit being composed of an extracellular domain (ECD) folded into a β-sandwich, a transmembrane domain (TMD) containing four α-helical strands (M1 to M4), and an intracellular domain (ICD) between M3 and M4, whose structure has partly remained elusive (*4*). The ion transmembrane pore is lined by the M2 helices of the five subunits and displays a strong selectivity for anions over cations (*5*, *6*). The main selectivity filter is thought to be located within the ion pore at the cytoplasmic end since mutations at this level are sufficient to convert GlyR-α1 into a cationic channel (*7*). Consistently, the chimeric construct obtained from the fusion of the ECD of the cation-selective channel *Gloebacter violaceus* pentameric ligand gated ion channel (GLIC) with the TMD of the anionic channel GlyR-α1 displays anionic selectivity with a unitary conductance identical to that of the full-length GlyR-α1 (*8*). Glycine-activated currents display multiple conductance states (86, 64, 46, 30, and 18 pS), with the predominant one (71%) being at 86 pS (*9*).

At the structural level, GlyR is among the best-characterized pLGIC, since several high-resolution structures in complex with modulatory ligands and in different conformational states have been deposited (*4*, *10*–*14*). Among them, multiple structures have been annotated as representative of the open, ion-conductive state. An early “wide-open” structure of the zebrafish GlyR-α1 determined by cryo–electron microscopy (cryo-EM) with detergents (*4*) displays an ion pore with a minimum diameter of 8.2 Å, which is inconsistent with the diameter of 5.3 Å estimated by electrophysiological recordings of polyatomic anions (*5*). By relaxing the wide-open conformation in room-temperature molecular dynamics (MD) simulations within a native lipid environment, we isolated a distinct open-channel state with a minimum pore diameter of 5.0 Å (*15*). This open state, referred to as “MD-open,” was stable, ion conductive, and anion selective in semiquantitative agreement with experiments (*16*). During the preparation of this report, a glycine and picrotoxin-bound (GlyR-Gly/PTX) structure of the zebrafish GlyR-α1 was solved in lipid nanodiscs and assessed as an open state by MD simulations with correct anionic selectivity and a computed conductance of 20 pS (*14*). Another GlyR-α1 structure in complex with glycine was published last year using the styrene maleic acid polymer (SMA) strategy (*4*). This structure, referred to as “GlyR-SMA-gly-open,” displays a minimum pore diameter of 5.6 Å, consistent with an ion-conductive state.

While the transmembrane pore allows for the selective translocation of ions across the membrane, chloride permeation in GlyR also involves the translocation of ions through the ECD and ICD, which contributes to the overall efficacy (conductance) of the channel. Although complete suppression of the ICD has minimal (if any) effect on the conductance (*17*–*19*), mutations of R377, K378, K385, and K386 in the putative MA stretch of GlyR-α1 into negatively charged glutamates cause reduction of the single-channel conductance up to 35%, with a more marked effect on inward currents (i.e., when chloride transits from the cytoplasm to the extracellular medium) (*20*). Mutations in the ECD that correspond to charge reversal or charge annihilation, such as K104A/G105D and D57I/R59T, reduce the outward conductance up to 70% (*21*). The anomalous mutation K104E by Moroni *et al.* (*22*) entails an asymmetric decrease of the conductance in both homomeric and heteropentameric GlyR, i.e., 22% of inward currents and up to 72.4% of outward currents, highlighting a marked inward rectification behavior. To rationalize these findings, it was proposed that residues located in the vestibule, i.e., K104, G105, and R59, contribute to a large and water-filled ion-conducting pathway in the ECD that mediates ion translocation via an apical opening (*23*).

To provide a description of the ion-permeation pathway(s) in GlyR with atomic resolution and explore the origin of rectification in its anomalous mutants, we performed a series of unbiased MD simulations of the “MD-open” model of GlyR-α1 in the presence of a transmembrane potential of varying strength and direction. Analysis of 469 chloride-permeation events on the wild type (WT) reveals that lateral fenestrations in the ECD, rather than the apical entrance, provide the main translocation pathway for chloride to reach the vestibule at the entrance of the ion transmembrane pore. The simulation results were used to construct a minimal kinetic model for chloride permeation that accounts for both conductance and rectification in GlyR WT and anomalous mutants. In silico electrophysiology of a series of mutations bordering the lateral fenestrations predict marked decreases in conductance, which we validate by single-channel patch-clamp electrophysiology. Our results provide the first complete description of the ion permeation pathway(s) in a prototypical pLGIC. The significance of these findings on the regulation of GlyR function and other pLGICs is discussed.

## RESULTS

### Computational electrophysiology of the MD-open model

**Fig. 1.**
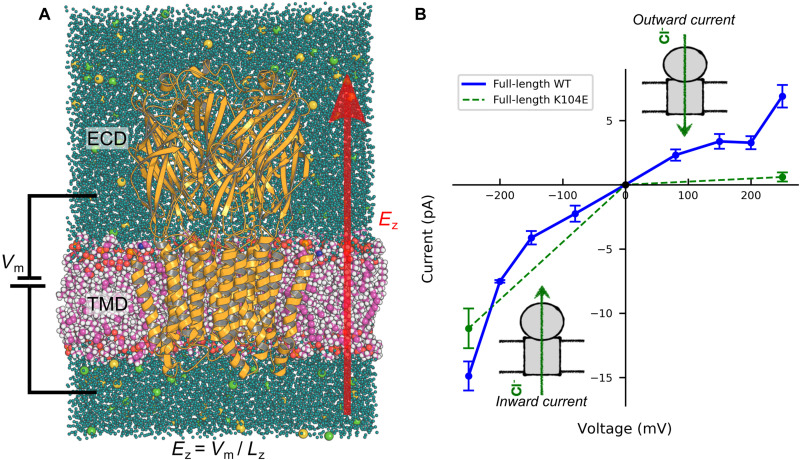
Computational electrophysiology of GlyR. (**A**) Atomistic representation of the simulation box with GlyR (orange) embedded in a POPC lipid bilayer (pink), water-solvated (light blue), and in the presence of 150 mM NaCl (yellow/green). The coordinates of the protein correspond to the MD-open state captured by Cerdan *et al.* (*15*). Our computational electrophysiology setup consists in the application of a constant electric field perpendicular to the membrane plane (red arrow). The resulting transmembrane potential, *V*_m_, is proportional to the strength of the electric field, *E*_z_, and the size of simulation box along the *z* axis, *L_z_*. Overall, the simulated molecular system includes ~220k atoms. (**B**) Current-voltage (*I*-*V*) curve obtained from computational electrophysiology of WT (blue) and the anomalous mutant K104E (dashed green). Each data point corresponds to the average current measured from a series of replicas simulated at one transmembrane potential. Currents were computed by counting the number of ion translocation events across the membrane per unit of time. Error bars were estimated assuming a Poisson distribution of the permeation events, i.e., σ = *I*/√*N*, with *I* being the current and *N* being the number of permeation events.

In a previous study (*15*), we investigated chloride permeation in the isolated TMD of the zebrafish GlyR-α1 embedded in a lipid bilayer. To maintain this “TMD-only” model in an open and conductive conformation, we applied harmonic restrains on the protein backbone. Here, we carry out a similar analysis using the cryo-EM construct of Du *et al.* (*10*) from zebrafish GlyR-α1 in the MD-open conformation, which is devoid of the ICD but includes the ECD and does not require the use of harmonic restraints (see Fig. 1A).

To investigate the mechanism of chloride permeation, we performed computational electrophysiology in 150 mM symmetrical sodium chloride at transmembrane potentials from −250 to +250 mV for a total simulation time of 13.9 μs (see Table 1 and table S1). As done in Cerdan *et al.* (*15*), the transmembrane potential was modeled using a constant electric field perpendicular to the membrane plane (see Materials and Methods). Overall, 13 to 173 chloride-translocation events across the membrane were collected at each voltage, yielding a statistically meaningful representation of the chloride flux through the channel over a broad range of experimental conditions. The results in Fig. 1B (blue) show that at physiological conditions (i.e., −150 < ∆*V* < + 150 mV), the current-voltage (*I*-*V*) relationship is almost linear, yielding a single-channel slope conductance of ~27 pS in agreement with single-channel recordings of GlyR-α1 from zebrafish and human that show similar conductances, i.e. 19 to 90 pS (*9*, *18*, *22*, *24*, *25*). Perhaps unexpectedly, outside the physiological range, the channel conductance is nonlinear with voltage, showing marked deviations at both positive and negative voltage. Intriguingly, when the same analysis was repeated on the TMD-only system, no rectification was observed (fig. S1).

**Table 1.** Computational electrophysiology. The experiments carried out on the GlyR-α1 cryo-EM construct (i.e., devoid of ICD) in the WT and the K104E mutant are presented. Numerical results on the ion translocating current, which correspond to the number of chloride permeation events cumulated over multiple simulation runs, are given in table S1. All MD simulations were produced in the presence of a 150 mM symmetrical concentration of NaCl.

**Voltage (mV)**	**−250**	**−200**	**−150**	**−80**	**80**	**150**	**200**	**250**	**−250 K104E**	**250 K104E**	**Total**
Cumulative simulation time (ns)	2045	1215	2520	926	2077	1663	2058	1442	1168	804	15,918
No. of independent runs	10	10	6	4	10	6	6	10	6	6	74

### Existence of a central vestibular cavity accessible through five lateral fenestrations and a single apical entrance

**Fig. 2.**
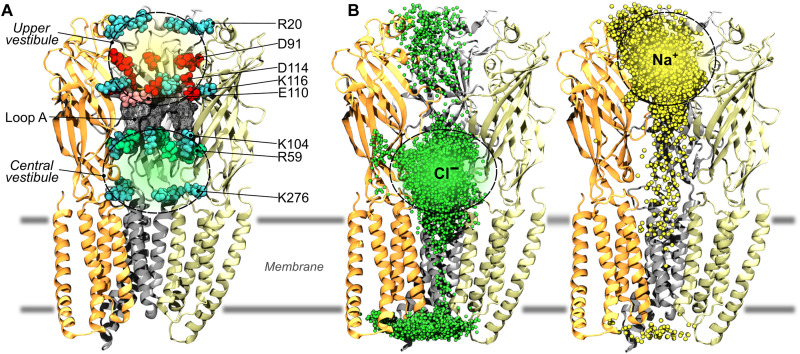
Identification of a central vestibular cavity in the ECD of GlyR that concentrates chloride at the entrance of the ion-transmembrane pore. (**A**) Amino acid composition of the vestibule. Charged residues located within 2.5 Å of sodium and chloride ions in the vestibule are represented as vdW spheres (i.e., the positively charged lysine and arginine residues are in cyan and green, respectively, whereas the negatively charged aspartic and glutamic acid residues are in red and pink, respectively). The gray mesh corresponds to residues (G105-H109) protruding from loop A, which form a constriction between the upper and lower portions of the vestibule. (**B**) Cumulated chloride (green) and sodium (yellow) ionic densities sampled by all-atom MD at zero voltage. The distributions clearly show that the vestibule is split into two water-filled compartments that promote an effective separation of ions with chloride ions predominantly occupying the lower compartment, here referred to as the central vestibule.

**Fig. 3.**
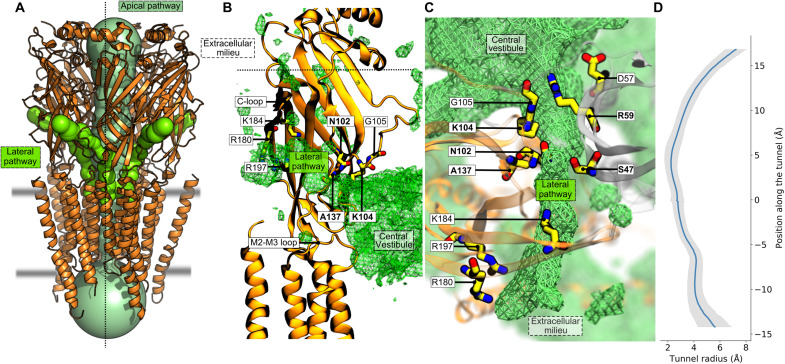
Lateral fenestrations connect the extracellular milieu with the central vestibule for chloride translocation in GlyR. (**A**) Channels and tunnels detected with the webserver MOLEonline on the GlyR-α1 cryo-EM structure (PDB: 6PM6). The classical vertical pathway for chloride translocation (apical) is shown in dark green. The previously unidentified lateral pathways that run almost parallel to the membrane and connect to the vestibule via openings at the subunit-subunit interfaces are shown in light green. (**B**) Sectional view of the lateral fenestrations with an indication of the lining residues. In green, the chloride density recorded over multiple simulation runs with a transmembrane potential of −250 mV. The lining residues that were mutated in silico and tested by computational electrophysiology are represented in licorice. Bold names indicate residues found to modulate channel conductance upon mutation in silico. (**C**) Top view of the chloride pathway via the lateral fenestrations with a section just below the C-loop. The green mesh represents an iso-chloride density. (**D**) Pore radius along the lateral pathways aligned with the structural representation in (C). The Hole profile was computed with Wordom. The blue line represents the average radius over multiple simulation replicas. The gray area corresponds to ± the SD. Note that the constriction point of these lateral translocation pathways overlaps with residues (+)-N102, (+)-A137, and (−)-S47; + and − indicate residues from the principal or the complementary subunit, respectively.

To extract information on the ion translocation pathway(s), the cumulated distributions of sodium and chloride ions were visualized during a 1-μs MD simulation with no voltage applied. As shown in Fig. 2, the simulations highlight that the extracellular cavity corresponding to the channel vestibule is split into two water-filled compartments that promote an effective separation of ions within the ECD, with chloride and sodium occupying predominantly the lower and the upper regions, respectively. These lower and upper compartments, here referred to as the central and the outer vestibule, respectively, are separated by a ring of amino acids (residues 105 to 109, GAHFH motif) that produce a physical constriction. Moreover, at both sides of the constriction, the compartments are lined by charged amino acids, which include rings of R20, D91, and D114-K116-E110 in the outer vestibule, and rings of K104-R59 and K276 in the central vestibule (see Fig. 2). Unexpectedly, chloride ions were also found in between the central vestibule and the EC (extracellular) medium, suggesting the existence of lateral fenestrations at the subunit interface for chloride translocation to the vestibule. To explore this hypothesis, the chloride density sampled by MD at −250 mV was analyzed in greater detail. As shown in Fig. 3, chloride-filled lateral fenestrations appear as narrow tunnels running approximately parallel to the membrane and opening to the EC medium right below the orthosteric glycine-binding site. Among the five interfaces, the widest tunnel has a diameter of 6 to 8 Å and is lined by positively charged residues at both the entrance (R59 and K104) and the exit (R180, K184, and R197) and by hydrophobic residues in the middle. Of note, N102 and A137 from the principal subunit and S47 from the complementary subunit form the constriction point of these fenestrations for chloride ions with a minimum diameter of 5 Å (Fig. 3D) that is comparable in size with the lumen of transmembrane pore. To investigate whether similar openings exist in the most recent cryo-EM structures of GlyR, we visualized the protein cavities of the GlyR-SMA-gly-open state using the web server MOLE*online* (https://mole.upol.cz) (*26*). The result reveals the existence of six pathways connecting the EC milieu and the central vestibule (Fig. 3A). Together, the simulations highlight the existence of a central vestibular cavity that is connected to the EC solution via six narrow tunnels, i.e., five lateral fenestrations between subunits and one apical pathway.

### Lateral fenestrations provide the main pathway(s) for chloride translocation between the EC medium and the central vestibule in the WT receptor

In addition to 469 ion permeation events across the membrane, the combined MD simulations sampled 748 chloride translocations between the EC medium and the central vestibule in both inward and outward directions. Among them, only 4% (31 events) of the total chloride flux proceeds via the apical pathway. Therefore, the lateral fenestrations provide by far the main chloride permeation pathway (96% or 717 events) to the central vestibule. To probe the existence and the functional relevance of lateral fenestrations in other GlyR structures, chloride permeation was probed by MD simulations with no transmembrane potential (i.e., at zero voltage), starting with the most recent cryo-EM structures annotated as representative of the active or the desensitized state, i.e., “GlyR-SMA-gly-open” α1 [Protein Data Bank (PDB): 6PM6], GlyR α2β (PDB: 5BKF), and the x-ray structure of GlyR α3 (PDB: 5VDH). In all cases, lateral fenestrations appear open and conductive with permeation rates comparable to those recorded in the MD-open conformation of the zebrafish GlyR-α1, i.e., 0.07 ± 0.02, 0.06 ± 0.04, and 0.07 ± 0.01 permeation/ns in GlyR α1, α2β, and α3, respectively, versus 0.04 ± 0.01 permeation/ns in MD-open (table S3).

### MD simulations recapitulate the rectification phenotype of K104E that lines the lateral fenestrations

The simulation results above predict that mutations in the lateral fenestrations could alter the GlyR conductance. Such a phenotype was already reported for mutations of K104 (*21*, *22*) and R59 (*21*), which line the lateral fenestrations, with the K104E mutant showing an inward-rectification behavior (*22*). To explore the origin of such an anomalous phenotype, the mutation K104E was introduced in the MD-open model and analyzed by computational electrophysiology. The results in Fig. 1 (green data points) are in quantitative agreement with the electrophysiological data showing 91% outward-current and 25% inward-current reductions relative to WT. The fraction of apical translocation events in the mutant increases from 4 to 67% at −250 mV (table S1). These observations thus indicate that chloride translocation via the lateral fenestrations is strongly hindered in the K104E mutant and becomes rate limiting on the outward current (i.e., chloride influx). Since K104E has a weaker effect on the inward current (i.e., chloride efflux), this mutation results into inward rectification. Therefore, the simulations highlight the critical role of the lateral fenestrations on chloride permeation in GlyR and account for the electrophysiological data of the anomalous mutant K104E.

### A minimal two-step translocation kinetic model qualitatively accounts for the K104E rectification mechanism

**Fig. 4.**
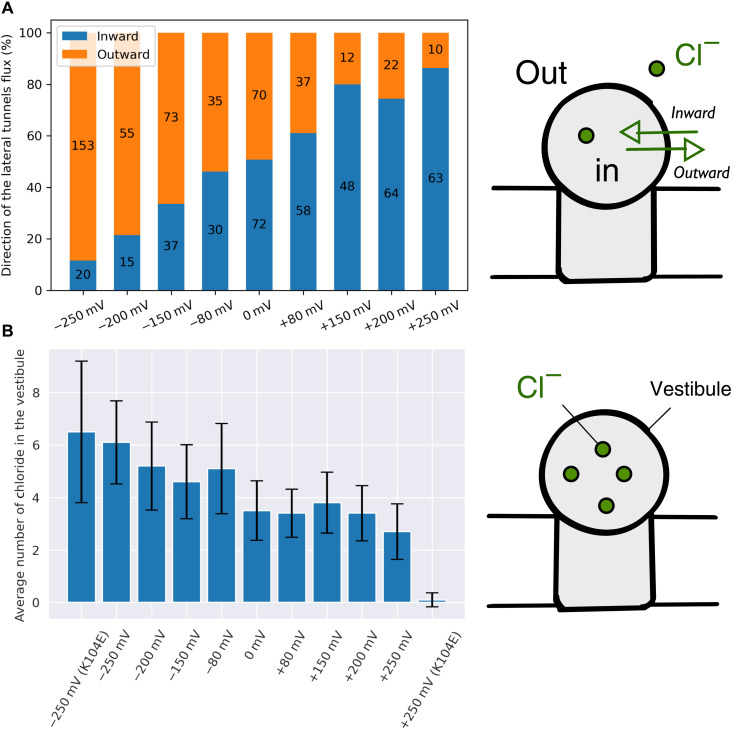
Chloride translocation via the lateral fenestrations of GlyR. (**A**) Chloride permeation via the lateral fenestrations is reversible at all voltages. The fraction of inward versus outward translocation is indicated in blue and orange, respectively. In the bars, the number of translocation events is given. At zero transmembrane potential, the inward and outward fluxes are in equilibrium. However, the inward/outward permeation ratio varies by two orders of magnitude from −250 to +250 mV. (**B**) The vestibular concentration of chloride is voltage dependent. The average number of chloride ions in the vestibule as measured from simulations at positive and negative voltage is given for WT and K104E. The data show that at negative voltages, chloride ions are pumped into the vestibule, whereas at positive voltages, the vestibular chloride is depleted.

The simulations suggest that the rectification phenotype in the anomalous mutant K104E is linked to the architecture of the receptor, i.e., the existence of a central vestibule connecting the extracellular milieu with the cytosol via narrow pores or tunnels (i.e., the apical pathway, the lateral fenestrations, and the transmembrane pore). In addition, they reveal that (i) chloride translocation through the lateral fenestrations is more frequent than permeation across the membrane and it is bidirectional (i.e., reversible) at all transmembrane potentials (Fig. 4A); (ii) chloride translocation across the membrane is bidirectional at zero voltage but becomes quasi-unidirectional (i.e., irreversible) at higher transmembrane potentials; (iii) the average number of chloride ions in the vestibule is about four at physiological conditions [(−200 : + 200) mV], but it increases to six at −250 mV and decreases to three at +250 mV (Fig. 4B).

**Fig. 5.**
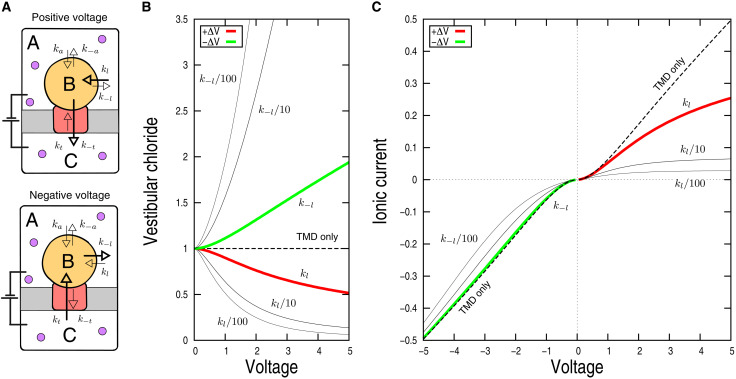
Minimalistic kinetic modeling of chloride permeation through GlyR WT and anomalous mutants. (**A**) The model. Chloride permeation is modeled here as a two-step translocation process involving three compartments (i.e., the extracellular medium, the central vestibule, and the intracellular medium) separated by two layers of barriers. Each translocation step is considered as reversible (see the main text). Here, *k_l_* and *k_a_* are kinetic rate constants for lateral and apical translocations to the vestibule, *k_t_* is the rate constant for translocation across the membrane; negative subscripts indicate translocation from the vestibule. In this model: *k_l_* = *k*_−*l*_ = 0.5, *k_a_* = *k*_−*a*_ = 0.025, and *k_t_* = *k*_−*t*_ = 0.1. (**B**) Vestibular concentration of chloride as a function of voltage at positive (red) and negative (green) transmembrane potentials. Values are normalized relative to the extracellular concentration of chloride as [*B*]*_SS_*/*A*_0_ (see Supplementary Text). Black thin lines correspond to mutants with hindered ionic translocations through the lateral fenestrations, i.e., *k_l_*/10 or *k_l_*/100. (**C**) Calculated *I*-*V* curve. Using the results of eqs. S5 and S7, *I*-*V* curves for the WT receptor (red/green) and the anomalous mutants (black) at both positive and negative voltages were obtained.

To explore the origin of rectification, the kinetics of chloride permeation were modeled using a two-step translocation process involving three compartments, i.e., the extracellular and intracellular milieu with same ion concentrations and the central vestibule, separated by two layers of energy barriers (Fig. 5A). For the modeling, we assumed that all chloride permeation events are reversible and that the translocation rate across the membrane is linear with voltage along the electrochemical gradient (*27*), while it decays exponentially with voltage in the reversed direction (i.e., against the electrochemical gradient). In addition, we assumed that chloride translocation via the lateral and apical pathways is voltage independent. In the limit of these hypotheses, we solved the model analytically to yield expressions for the vestibular concentration of chloride and the net translocating currents at steady state (see Supplementary Text). The results are summarized below.

At positive voltage (chloride influx), chloride ions are pumped out of the vestibule via the ion transmembrane pore and filled back via the lateral and apical pathways. Since chloride translocation across the membrane increases linearly with voltage, while the lateral and apical permeations are not, the model predicts that chloride is depleted from the vestibule with increasing voltage (Fig. 5B, red line) until the net chloride flux is limited by the lateral or apical translocation rates. If so, at large positive voltage, chloride translocation across the membrane becomes voltage independent (eq. S8 and Fig. 5C, red line), thereby decreasing the channel conductance. In addition, the model predicts that if the permeability of the lateral portals is reduced, e.g., by mutations that hinder or partly occlude chloride translocation, the net translocating current will decrease until the lower bound imposed by the apical permeation is reached (Fig. 5C, black lines). Hence, simple kinetic modeling suggests that strong deviations from linearity of the *I*-*V* curve at positive voltage, in particular for K104E (*22*), are due to the existence of additional barriers along the chloride permeation pathway(s) that are essentially voltage independent.

At negative voltage (chloride efflux), chloride ions translocate to the central vestibule via the ion transmembrane pore and exit it through the lateral and apical pathways (Fig. 5A). Assuming that the rate constants for lateral and apical chloride translocations are voltage insensitive, the model predicts that the vestibular concentration of chloride increases with voltage and may reach levels higher than extracellularly, i.e., chloride ions are pumped into the vestibule (Fig. 5B, green line). Nonetheless, the model predicts that the deviation from a linear *I*-*V* relation is weaker than at positive voltage because chloride pumping to the vestibule accelerates ion permeation through the lateral and apical pathways by a concentration effect and the unitary conductance will be only marginally affected (Fig. 5C, green line). Insightfully, the kinetic equations predict that the smaller but detectable deviation from Ohm’s law at negative voltage in the anomalous mutant K104E (*22*) is due to a nonnegligible chloride flux against the electrochemical gradient in the low-voltage range. This effect disappears in the limit of large transmembrane potentials when chloride translocation across the membrane becomes essentially irreversible (eq. S12).

Hence, simple kinetic modeling provides evidence that rectification originates from the existence of multiple barriers along the ion permeation pathway with some of them being voltage insensitive. Moreover, the model predicts that mutations hindering or blocking ion translocation to the vestibule introduce asymmetries in the *I-V* curve consistent with patch-clamp electrophysiology of the anomalous mutant K104E.

### In silico analysis of mutations predicted to hinder lateral permeation

To further challenge the functional relevance of the lateral fenestrations for ion conductance in GlyR, several mutations were explored in silico. For this purpose, three positively charged residues at the periphery of the ECD (i.e., R180, K184, and R197), three neutral amino acids in the middle of the lateral pathways (i.e., N102, A137, and S47), and four residues at their vestibular end (i.e., D57, R59, K104, and G105) were considered for mutagenesis. Since known anomalous mutants, i.e., K104E, D57I/R59T, and K104A/G105D, involve charge reversal or annihilation, mutations promoting positive charge neutralization or negative charge addition were explored. In addition, taking inspiration from the concept of hydrophobic gating (*28*), neutral residues in the inner portion of the fenestrations were substituted by bulkier and more hydrophobic side chains (i.e., leucine, isoleucine, and phenylalanine) or mutated into negatively charged residues such as aspartate and glutamate.

**Fig. 6.**
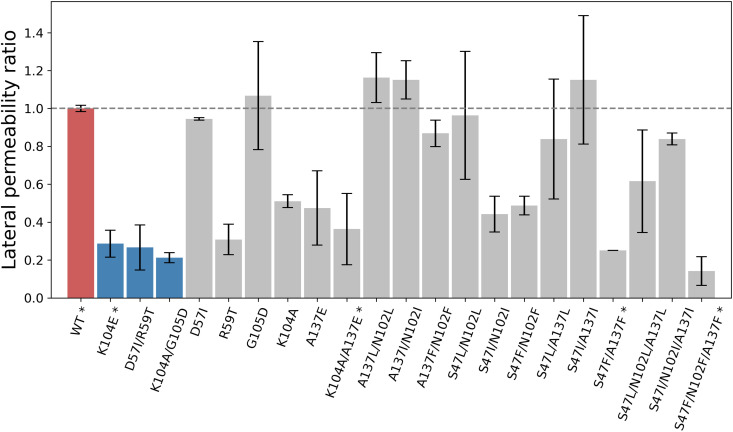
In silico screening of mutations that reduce chloride permeability via the lateral fenestrations. The lateral permeability ratio is shown for the WT (red), three ECD mutants from the literature that are known to reduce channel conductance (dark blue), and several mutations explored in silico in this work (gray). Lateral permeability ratios were determined by computational electrophysiology at a transmembrane potential of −280 mV and 150 mM concentration of NaCl; see Materials and Methods. The number of replicas and the simulation length per mutant are given in table S4. Lateral permeability ratios <1 indicate a reduction of the lateral permeability relative to the WT. Error bars were evaluated as SEM over two or more replicates. By setting a threshold of 0.4 in the lateral permeability ratio, which is consistent with numerical results obtained for three anomalous mutants from the literature (dark blue), the in silico screening prioritizes two double mutants and one triple mutant (marked by *), which were tested by single-channel electrophysiology in this work.

Nineteen single-point mutations were introduced in the full-length receptor and corresponding mutants explored by in silico electrophysiology at −280 mV. To characterize the mutant phenotype, lateral permeability ratios were computed and compared (see Materials and Methods). The results highlight that the introduction of a negative charge within the vestibule (i.e., A137E or S47D) may promote a substantial reduction of the lateral permeability ratio akin the anomalous mutant K104E (fig. S2). At the same time, mutations that do not involve changes in the electrostatic potential of the vestibule (i.e., A137L or S47I) may have nonnegligible effects. Synergistic effects of multiple hydrophobic substitutions were also explored. In contrast to single-point mutations, double and triple mutants produced much stronger reductions of the lateral permeability ratio (Fig. 6). In particular, the S47I/N102I and S47I/N102F mutations decreased the lateral permeability ratio by 50% (0.44 ± 0.13 and 0.49 ± 0.07, respectively), S47F/A137F by 75% (0.25 ± 0.0), and the triple phenylalanine mutant S47F/N102F/A137F by 86% (0.14 ± 0.11). Last, the A137E mutation was explored in combination with K104A. The double mutant K104A/A137E reduced the lateral permeability by 64% (0.36 ± 0.27), which is more pronounced than the effect introduced by either mutation alone, i.e., 42 and 49% reduction for A137E and K104A, respectively (see Fig. 6). Therefore, the simulations predict that charge reversal at the vestibular end of the lateral fenestrations, i.e., A137E/K104A, or hydrophobic substitutions in their inner region, i.e., S47F/A137F or S47F/N102F/A137F, profoundly affect the permeability of the ECD to chloride, thereby affecting channel conductance. These three mutants were engaged in experimental testing.

### Single-channel analyses of mutants predicted in silico establish the key contribution of lateral fenestrations to chloride translocation in human α1 GlyR

Outside-out single-channel recordings were performed on human embryonic kidney (HEK) 293 cells transiently transfected with the full-length human GlyR-α1 using symmetrical ionic concentrations at various potentials from −100 to +100 mV with a step interval of 20 mV (see Materials and Methods). Currents were recorded for the WT receptor and the three anomalous mutants K104E, K104A/A137E, and S47F/A137F [see tables S7 and S6 for the determination of the median effective concentration (EC_50_) of these mutants]. The triple mutant S47F/N102F/A137F was also explored but yielded no significant current.

**Fig. 7.**
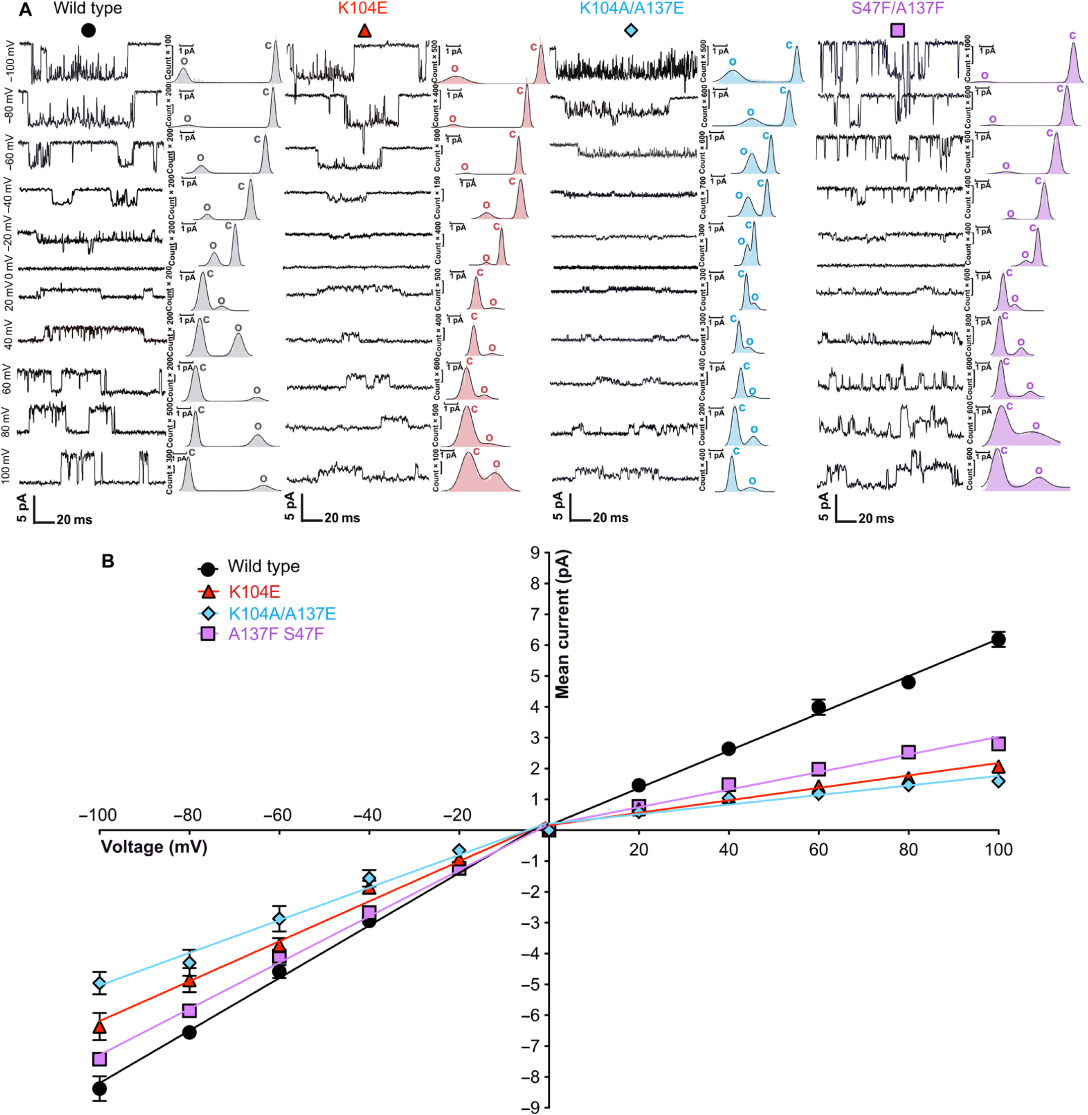
Single-channel electrophysiology. (**A**) Representative recordings of single-channel outside-out currents for the four constructs transiently transfected in HEK-293T cells explored at different voltages toward application of 1 to 10 μM glycine. Data points are color-coded as follows: WT GlyR-α1 in black/circle; K104E in red/triangle; K104A/A137E in blue/diamond; S47F/A137F in purple/square. Recordings were performed in symmetrical chloride solutions. Histograms of mean currents are also represented (panel on the right for each construct) with c representing the closed-state channel and o the open-state channel. (**B**) *I*-*V* relationships were obtained by plotting the mean current versus the applied voltage with SEM as error bars. Negative-voltage and positive-voltage data points were fitted by lines separately to compare slope conductance at positive and negative voltages and evaluate the rectification index. For each point, results from *n* = 5 to 7 cells were combined (except WT at +100 mV is *n* = 4, K104A/A137E at +100 mV is *n* = 4, and K104E at +100 mV is *n* = 2). Inward and outward slope conductance values are as follows: ○ WT: 85.16 and 60.47 pS; △ K104E: 64.78 and 20.19 pS; ◇ K104A/A137E: 52.89 and 15.52 pS; and □ S47F/A137F: 74.82 and 28.52 pS (see also Table 2 and table S7).

The WT receptor displays *I-V* relationships at negative and positive voltage that can be fitted by two linear slopes, yielding an inward (slope) conductance of 85 pS and an outward (slope) conductance of 60 pS, consistent with previous work (*21*, *22*) (see Fig. 7B and Table 2). The rectification index for WT, here 1.41 for inward/outward slope, is in close agreement with the work of Moorhouse *et al.* (*24*). For K104E, we measured a slope conductance of 64 pS (inward) and 20 pS (outward), highlighting a stronger effect of the mutation on the chloride influx. Of note, the outward slope conductance measured for the K104E mutant was 19.8 ± 0.7 pS in Moroni *et al.* (*22*), while we found 20.19 ± 0.91. However, the inward slope conductance, i.e., 85.16 ± 2.584 pS (WT) and 64.78 ± 3.25 pS (K104E), were different from those measured by Moroni *et al.* (*22*), i.e., 47.3 ± 2.1 pS (WT) and 36.9 ± 0.6 pS (K104E), although the relative change upon mutation was remarkably similar, i.e., 22% reduction in Moroni *et al.* (*22*) and 24% here. Discrepancies at negative voltage can be explained considering that Moroni *et al.* (*22*) did not use the same solutions for recording at negative or positive voltages, and their experiments were carried out in the cell-attached configuration.

**Table 2.** Single-channel electrophysiology. Inward and outward slope conductance values (**Ɣ**) and corresponding rectification index calculated for GlyR-α1 WT and mutants expressed in HEK293 cells and recorded in outside-out configuration. Statistical significances (*P* values) were obtained by one-way ANOVA followed by Dunnett’s multiple comparison test (compared to WT): *P > 0.05, **P > 0.01, ***P > 0.001 (see also table S7).

**Receptor construct**	**Inward Ɣ (slope at negative potential) (pS)**	**Outward Ɣ (slope at positive potential) (pS)**	**Calculated rectification index (inward slope Ɣ/outward slope Ɣ)**
Wild type	85.16 ± 2.584	60.47 ± 2.005	1.41
K104E	64.78 ± 3.25*	20.19 ± 0.91**	3.2
K104A/A137E	52.89 ± 3.598***	15.52 ± 1.476***	3.41
S47F/A137F	74.82 ± 1.475	28.52 ± 1.453**	2.62

On K104A/A137E, we measured a reduction of 74% in the outward conductance and 38% in the inward conductance as compared to WT, yielding a rectification index of 3.41 that is threefold higher than that of WT (Table 2). On S47F/A137F, we measured a reduction of 12 and 53% in inward and outward ionic conductance, yielding a rectification index of 2.62 that is twofold higher than that of WT (Table 2). Together, these findings provide direct evidence that lateral fenestrations in the ECD are key determinants of chloride permeation in GlyR. Note that the anomalous mutants K104E, D57I/R59T, or K104A/G105D, and now K104A/A137E and S47F/A137F, which alter channel conductance without drastically affecting the gating process, can be classified as mutations of the γ phenotype (*29*).

## DISCUSSION

Ion permeation in response to increased levels of neurotransmitters is at the very core of signal transduction at chemical synapses. Using a synergistic combination of all-atom MD simulations and single-channel electrophysiology, we provide here unprecedented insights onto ionic translocation through the homomeric GlyR-α1 with possible implications for the pLGIC superfamily. Note that our simulation analysis was carried out using a receptor construct devoid of the ICD, so that its effect on ion permeation and/or rectification could not be accounted for. However, our experimental validation of the computational predictions was done in a full-length receptor including the ICD.

The spatial distribution of the chloride density map sampled by MD reveals the existence of a central vestibular cavity in the ECD of the receptor that is made accessible from the EC medium by five lateral fenestrations and one apical entrance. Statistics over chloride translocation in computational electrophysiology support the conclusion that the lateral fenestrations provide the main translocation pathway for chloride to enter and exit the vestibule. In addition, amino acid substitutions that introduce a negative charge (K104A/A137E) at the vestibular end of the lateral pathways or bulky and hydrophobic side chains (S47F/A137F) near their constriction point were found to alter the unitary conductance in single-channel electrophysiology. Together, the combined simulations and electrophysiology data demonstrate that the chloride flux within the ECD of GlyR-α1 is controlled by lateral fenestrations. This discovery provides an unanticipated pathway for chloride permeation that challenges the apical translocation model commonly accepted in pLGICs (*30*–*32*).

The inward rectification of the landmark mutant K104E by Moroni *et al.* (*22*) was reproduced here in simulations and single-channel electrophysiology. The existence of lateral pathways for ion translocation that open in correspondence of this lysine residue provides an alternative interpretation of the anomalous mutant phenotype. Our analysis suggests that the inward rectification of K104E is most likely due to a reduced permeability of chloride through these lateral pores in the ECD rather than a change in the electrostatics along the apical pathway. Consistent with this hypothesis, the newly identified S47F/A137F mutant was shown to produce a similar inward rectification with no change in the electrostatic potential of the vestibule. In addition, using a minimal kinetic model, we show that impairing the passage of chloride ions through the lateral fenestrations strongly decreases the vestibular concentration of chloride at positive voltage, thereby decreasing the unitary inward chloride conductance. On the other hand, the outward chloride conductance at negative voltage is predicted to be only marginally affected in the mutant because chloride pumping to the vestibule forces the passage of ions through both the lateral and apical pathways by a mechanism resembling a “pressure valve.” These predictions are consistent with the single-channel electrophysiology of the K104E mutant (*22*). More generally, our observations support the conclusion that inward rectification in this ion channel has a kinetic origin and is a direct consequence of the molecular architecture of the receptor featuring a reservoir (i.e., the central vestibule) that can be filled by or depleted of ions in a voltage-dependent manner. This architectural element introduces additional voltage-insensitive barriers along the chloride permeation pathway(s) that strongly reduce channel conductance when the vestibular chloride is depleted (positive voltage) and have only minor effects when chloride concentrates in the vestibule (negative voltage), thereby promoting current rectification. Intriguingly, a similar mechanism might explain why substitutions of positively charged residues (R377, K378, K385, and K386) at the ICD of GlyR-α1, which is likely to form another (smaller) cavity at the bottom of the receptor, strongly decrease the unitary conductance preferentially in the outward chloride direction (*20*). Note that the rectification mechanism emerging from this analysis involves no voltage-dependent channel block as established in glutamate-gated *N*-methyl-d-aspartate receptors or the inward-rectifier K^+^ channels (*33*, *34*), nor does it involve the inactivation ball for the N-type inactivation of voltage-gated channels (*35*), and it has not been previously reported. Voltage-dependent block by intracellular polyamine has been shown for neuronal nAChRs (nicotinic AcetylCholine Receptors) (*36*, *37*), causing inward rectification that can possibly occur together with a pressure valve type of rectification.

Several lines of evidence suggest that lateral fenestrations for ion permeation in the ECD exist and contribute to ion conductance in other pLGICs. First, sequence alignment of a set of pLGICs [figure S5 from (*38*)] shows that anionic channels carry an excess of positively charged residues near the vestibular end of the lateral fenestrations, whereas cationic channels feature a nearly conserved negatively charged aspartate. Second, amino acid substitutions at the position corresponding to K104 in GlyR-α1 with residues that annihilate (*23*) or revert the charge (*21*, *22*) strongly affect the unitary conductance in both anionic and cationic pLGICs [i.e., γ-aminobutyric acid type A receptor (GABA_A_R) K105, 5-HT_3_R D132, and muscle nAChR D97] without altering the charge selectivity (*39*). Third, lateral fenestrations have been already detected in GABA_A_R (*40*, *41*), 5HT_3_R (*42*), STeLIC (endosymbiont of *Tevnia jerichonana pentameric* ligand gated ion channel) (*43*), and ELIC (*Erwinia chrysanthemi* pentameric ligand gated ion channel) (*44*), and these observations are now extended to a larger set of pLGICs (table S8). Fourth, the existence of lateral fenestrations for ion conductance appears to overcome the problem of glycans in recent cryo-EM structures of GlyR α2β (*45*) and GABAR α1β1γ2 (*46*) or α1β3γ2 (*47*), where sugar moieties located at the apex of the receptor clearly hinder and possibly occlude the apical pathway. Fifth, MD simulations of 5-HT_3_R captured the spontaneous translocation of sodium ions via similar lateral tunnels along with an effective separation of anions/cations in the ECD (*42*). Last, a similar permeation mechanism has been described in trimeric channels, e.g., P2X receptors, based on modeling and cross-linking experiments (*48*, *49*). Together, functional, structural, and simulation studies support the conclusion that lateral fenestrations for ion translocation to the vestibule are relevant if not an absolute functional requirement for synaptic transduction by pLGICs.

The discovery that ion conductance in pLGICs involves the translocation of ions through narrow tunnels located >40 Å away from the transmembrane pore yields unprecedented mechanistic insight onto neurotransmitter receptors function that offers opportunities to control synaptic transduction allosterically. In this context, protein-receptor interactions hindering or occluding ion translocation to the vestibule or the application of drugs targeting protein cavities overlapping with the lateral fenestrations emerge as possible modulatory strategies. The pharmacological potential of the lateral fenestrations in GlyR and other pLGICs remains to be explored.

## MATERIALS AND METHODS

### Modeling of the active state

The relaxation of the glycine-bound, cryo-EM structure of GlyR-α1 (*10*) was repeated in its physiological environment using the protocol described in (*15*); the latter involves a room-temperature relaxation of the protein coordinates by explicit water/membrane MD in the presence of a symmetry restraint on the pentameric organization of the receptor and positional restraints on the backbone atoms that are progressively removed over 50 ns. At the end of the relaxation, a long equilibration run by unbiased MD was carried out for a total simulation time of 450 ns; see fig. S3. Analysis of the ion transmembrane pore during the last 50 ns by the program HOLE (*50*) indicates that the pore lumen at the constriction point is 2.67 Å with an SD of 0.32 Å. These results indicate that the MD-relaxed structure of GlyR-α1 is physically open to chloride; it is structurally consistent with the open-channel state isolated in our previous analysis (*15*, *16*); and it presents a pore lumen in excellent agreement with the experimental predictions based on permeability to polyatomic anions (*5*). In addition, the large fluctuations sampled by MD at the constriction point (fig. S4) highlight a highly dynamic behavior of the gate, which is likely to assist chloride permeation. On the basis of these these observations, we decided to randomly extract 10 snapshots of the protein structure from the last 50 ns of the trajectory, which were used to start the computational electrophysiology experiments.

### System preparation

PDB structures of the GlyR-α1 extracted from the initial trajectory (see above) were embedded in a POPC (1-Palmitoyl-2-oleoyl-sn-glycero-3-phosphocholine) membrane bilayer of 120 × 120 Å^2^, solvated with TIP3P (transferable intermolecular potential with 3 points) water molecules (22.5 Å at the top and the bottom of the protein), and solvated with 150 mM NaCl, using CHARMM scripts from CHARMM-GUI (*51*, *52*) but executed locally. Concerning the mutants of GlyR-α1, the mutations were introduced by modifying the name of the concerned residues in the initial PDB before following the same procedure of setup with CHARMM (*53*). The system prepared using these parameters are constituted of about 230k atoms and measured 120 × 120 × 170 Å^3^.

### MD simulations

All-atom MD simulations were run with GROMACS 2019.4 (*54*), using periodic boundaries conditions, and the CHARMM36 forcefield (*55*, *56*) with CHARMM36m modifications (*57*). The minimization and equilibration protocols used default parameters generated by CHARMM-GUI (*58*) for GROMACS. In short, after 5000 steps of energy minimization using the steepest-descent algorithm, the system was heated at 300 K by generating random velocities with the Berendsen thermostat for 50 ps using a 1-fs integration time step. Then, the system was coupled semi-isotropically to a Berendsen barostat and further equilibrated for 25 ps using a 1-fs time step, and 200 ps with a 2-fs time step. During the equilibration, atomic position restraints on the protein heavy atoms were gradually relaxed from 4000 and 2000 kJ mol^−1^ nm^−2^ for the protein backbone and side-chains, respectively, to 0. The production runs were carried out in the canonical ensemble at constant volume, temperature and number of particles (NVT) using a modified Berendsen thermostat (*59*) to maintain the temperature at 300 K and the Parrinello-Rahman barostat (*60*) to set the pressure at 1 bar. The LINCS algorithm was used to constrain bonds involving hydrogens (*61*) and particle mesh Ewald to treat the long-range electrostatic interactions (*62*).

### Computational electrophysiology

To quantify channel conductance in simulation, we introduced a constant electric field *E_z_* along the *z* direction that is perpendicular to the membrane plane. The resulting transmembrane potential *V_m_* is proportional to the strength of the electric field and the length of the simulated box *L_z_* as *E_z_ = V_m_/L_z_* (*63*). Estimates of ionic currents were obtained by counting the number of permeation events per unit of time using the FLUX module in Wordom (*64*, *65*). Error estimates δ on the calculated current *I* were obtained assuming a Poisson distribution of the permeation events such that δ = *I*/√*N*, with *N* being the number of permeation events (*66*). Last, channel conductance *g* was computed as *g = I/V_m_*. Ion permeation events through the lateral/apical pathways and the number of chloride ions in the vestibule were computed using in-house TCL scripts in VMD (*67*).

Computational electrophysiology of engineered mutants was carried out at a transmembrane potential of −280 mV. Although nonphysiological, the use of a large and negative voltage ensures significant amplitude of the ionic current even in the presence of mutations hindering chloride translocation (e.g., K104E in Fig. 1), which grants for meaningful statistics on the simulation time scale. The effect of the mutation was then quantified by measuring the fraction of the chloride flux exiting the vestibule laterally, here termed lateral permeability ratio, which was computed as the outward chloride permeation rate via the lateral fenestrations over the inward permeation rate across the membrane. This observable was found to be robust against sampling inefficiency as it normalizes the lateral permeation rate by the chloride translocation rate across the membrane, which may differ from replica to replica particularly at low voltage. This analysis carried out on the anomalous mutants K104E from Moroni *et al.* (*22*) and D57I/R59T and K104A/G105D from Brams *et al.* (*21*) provides lateral permeability ratios of 0.27 ± 0.17 and 0.21 ± 0.04 for D57I/R59T and K104A/G105D, respectively, and 0.29 ± 0.10 for K104E, which are significantly lower than 1.00 ± 0.02 for WT; see Fig. 6. Therefore, quantification of the lateral permeability ratio at strong and negative voltage provides a computationally efficient way to probe for the effect of mutations hindering ion translocation through the lateral fenestrations.

### Lateral tunnel automatic detection

We used the MOLE*online* webserver (https://mole.upol.cz) (*26*) to detect lateral tunnels within PDB structures of various conformational states of the receptor. We used default parameters but the following: Probe radius set to 40, Interior Threshold set to 1.4, Bottleneck Radius set to 1.4, Bottleneck Tolerance set to 0, and Max Tunnel Similarity set to 0.15.

### Molecular biology

Mutations were introduced into the α1 WT human glycine receptor subcloned in pmt3 vector using the CloneAmp HiFi PCR Premix kit of PCR (Takara). All introduced mutations were confirmed by DNA sequencing (Eurofins Genomics).

### Expression in cultured cells

HEK-293 cells were cultured in Dulbecco’s modified Eagle’s medium with 10% fetal bovine serum (Invitrogen) in an incubator at 37°C and 5% CO_2_. After being phosphate-buffered serum–washed, trypsin-treated (Trypsine-EDTA; Thermo Fisher Scientific), and seeded on petri dishes, cells were transiently transfected using calcium phosphate-DNA coprecipitation with glycine receptor constructs (2 μg DNA) and a construct coding for a green fluorescent protein (0.2 μg). One day after transfection, cells were washed with fresh medium, and recordings were carried out within 24 hours.

### Outside-out recordings

Recording currents are obtained with an RK-400 amplifier (BioLogic) using pClamp 10.5 software, digitized with a 1550 digidata (Axon instruments). Recording pipettes were obtained from thick-wall borosilicate glass (1.5 mm by 0.75 mm by 7.5 cm; Sutter Instrument) using a micropipette puller (P-1000, Sutter Instrument) and fire-polished with a micro-forge (MF-830, Narishige) to be used at resistances between 7 and 15 megohms. Micropipettes were filled with internal solution [that contain 152 mM NaCl, 1 mM MgCl_2_, 10 mM 1,2-bis(2-aminophenoxy)ethane-*N*,*N*,*N*′,*N*′-tetraacetic acid, and 10 mM Hepes, pH adjusted to 7.3 with NaOH solution, osmolarity measured at 335 mosM]. Extracellular solution (152 mM NaCl, 1 mM MgCl_2_, and 10 mM Hepes, pH adjusted to 7.3 with NaOH solution and osmolarity was adjusted to 340 mosM with glucose) was delivered by an automated perfusion system (RSC-200, BioLogic). Agonists’ solutions are freshly made before sessions of recordings and are obtained with extracellular solution added with 1 to 10 μM glycine (dissolved from stock solution of 1 M in water). Acquisition of recordings was performed at the sampling of 20 kHz and low-pass–filtered at 1 kHz (using the amplifier five-pole Bessel filter). For the establishment of *I-V* curves, voltage is first clamped at −60 mV. Sweeps of 10,000 ms are performed containing the following steps: voltage held at −60 mV in external solution (843.8 ms); voltage held at *x* mV in external solution (1000 ms); voltage held at *x* mV in external solution containing 1 to 10 μM glycine (4000 ms); voltage held at *x* mV in external solution (1000 ms); and voltage held at −60 mV in external solution (2843.7 ms). The *x* voltage is exchanged (−100, −80, −60, −40, −20, 0, +20, +40, +60, +80, and +100), allowing us to perform 11 sweep-long recordings for each outside-out patch. Openings are analyzed using Clampfit 10 software, and currents were calculated by fitting the all-points histogram distributions of current amplitudes with the sum of two Gaussian curves. No further filtering is performed for the analysis. Outside-out recording analyses were obtained for the 11 sweeps (referring to each voltage) in the same outside-out patch if not otherwise stated.

### Expression in oocytes

Ovarian fragments from *Xenopus laevis* (European Xenopus Resource Centre, Portsmouth) were bathed into ORII solution (82.5 mM NaCl, 2.5 mM KCl, 1 mM MgCl_2_, and 5 mM Hepes, pH adjusted to 7.6 with NaOH). Enzymatic treatment is performed by collagenase II (1 mg/ml; 1 hour at room temperature in gentle agitation) to isolate oocytes and remove follicular membranes. Oocytes were then selected and bathed into Barth’s medium [88 mM NaCl, 1 mM KCl, 0.33 mM Ca(NO_3_)_2_, 0.41 mM CaCl_2_, 0.82 mM MgSO_4_, 2.4 mM NaHCO_3_, and 10 mM Hepes] at 18°C. WT glycine receptor and mutant DNAs were injected (10 ng) with a fluorescent green protein containing vector (25 ng) in the oocyte nucleus. Fluorescent oocytes were then recorded 48 to 72 hours after injection.

### TEVC in oocytes

Oocytes were placed in a recording chamber and perfused with ND96 solution (96 mM NaCl, 2 mM KCl, 5 mM Hepes, 1 mM MgCl_2_, and 1.8 mM CaCl_2_, freshly made, adjusted at pH 7.6 with NaOH concentrated solution) and glycine-containing solutions. Micro-electrodes of resistances comprised between 0.2 and 2 milliohms (pipette puller PC-10, Narishige) were used, and oocytes were clamped at −60 mV. Recordings are performed with an oocyte clamp OC-725C amplifier (Warner Instrument Corp.), digitized with an AxonInstrument 1550 and pClamp software. Analyses were made with Clampfit (Molecular Devices, Sunnyvale, CA). Dose-response curves, EC_50_, and Hill coefficients are obtained by the normalization of glycine-induced currents followed by the fitting of curves by one-site Hill equation (GraphPad Prism).

## Supplementary Materials

### This PDF file includes:

Supplementary Text Figs. S1 to S5 Tables S1 to S8 References

## Notes

View/request a protocol for this paper from *Bio-protocol*.
