# Supplementary material for: Lateral fenestrations in the extracellular domain of the glycine receptor contribute to the main chloride permeation pathway
Source: Sci Adv. 2022 Oct 14;8(41):eadc9340. doi: 10.1126/sciadv.adc9340 (PMC9565810; doi:10.1126/sciadv.adc9340)
Supplement: Supplementary file 1 — Supplementary Text Figs. S1 to S5 Tables S1 to S8 References [file sciadv.adc9340_sm.pdf]

Science Advances
AAAS

Supplementary Materials for

Lateral fenestrations in the extracellular domain of the glycine receptor contribute to the main chloride permeation pathway

Adrien H. Cerdan *et al.*

Corresponding author: Marco Cecchini, mcecchini@unistra.fr;
Pierre-Jean Corringer, pierre-jean.corringer@pasteur.fr

Sci. Adv. **8**, eadc9340 (2022)
DOI: 10.1126/sciadv.adc9340

This PDF file includes:

Supplementary Text
Figs. S1 to S5
Tables S1 to S8
References

Supplementary Text

Kinetic modeling of ion translocation

To explore the origin of rectification, chloride permeation was modeled as a two-step translocation process involving three compartments separated by two layers of energy barriers; see **Fig. 5A**. Assuming reversible ionic translocation between the central vestibule and both extracellular and intracellular solutions, chloride permeation through GlyR can be modeled as

$$A \underset{k_{-l}+k_{-a}}{\overset{k_l+k_a}{\rightleftarrows}} B \underset{k_t}{\overset{k_{-t}}{\rightleftarrows}} C \qquad \text{(A.1)}$$

where k_l and k_a are the kinetic rate constants for lateral and apical ionic translocations to the central vestibule, respectively, and k_t is the rate constant for chloride translocation across the membrane; the minus sign in the subscript is used for translocation away from the vestibule. Given an initial concentration of chloride in the extracellular and intracellular media at symmetric ionic conditions (A_0) and values for all rate constants in Eq. A1, the time-dependent vestibular concentration of chloride is obtained by solving the first-order differential equation

$$\frac{d[B]}{dt} = k_t A_0 - k_{-t}\, B(t) + (k_l + k_a)A_0 - (k_{-l} + k_{-a})\, B(t) \qquad \text{(A.2)}$$

By imposing $[B] = 0$ at $t = 0$ as a boundary condition, it yields

$$B(t) = \frac{k_l + k_a + k_t}{k_{-l} + k_{-a} + k_{-t}}\left[1 - e^{-(k_{-l}+k_{-a}+k_{-t})}\right]A_0 \qquad \text{(A.3)}$$

In the limit for $t \to \infty$, Eq. A3 yields the vestibular concentration of chloride at steady state

$$[B]_{SS} = \frac{k_l + k_a + k_t}{k_{-l} + k_{-a} + k_{-t}}A_0 \qquad \text{(A.4)}$$

At positive voltage (inward chloride flux), assuming that the rate constant for outward versus inward chloride translocation across the membrane decays exponentially with voltage, i.e., $k_t = k_{-t}\exp(-\beta\Delta V)$, Eq. A4 yields

$$[B]_{SS} = \frac{k_l + k_a + k_{-t}\, e^{-\beta\Delta V}}{k_{-l} + k_{-a} + k_{-t}}A_0 \qquad \text{(A.5)}$$

with β being the inverse temperature $1/kT$. Assuming that the inward permeation rate increases linearly with voltage as in the Nernst-Planck equation (27), in the limit of large transmembrane potentials (i.e., $k_{-t} \gg k_{-a}, k_{-l}$ and $e^{-\beta\Delta V} \to 0$) Eq. A5 reduces to

$$[B]_{SS} = \frac{k_l + k_a}{k_{-t}}A_0 \qquad \text{(A.6)}$$

which predicts that at positive voltage the vestibular concentration of chloride is voltage dependent as $k_{-t} \propto \Delta V$ and depends on both lateral and apical translocation rates. Using the result of Eq. A6 and assuming bidirectional chloride flux across the membrane (i.e., $i_+ = k_{-t}[B]_{SS} - k_t A_0$), an expression for the net translocating current at positive voltage is obtained

$$i_+ = \left(\frac{k_l + k_a + k_{-t}\, e^{-\beta \Delta V}}{\frac{k_{-l}}{k_{-t}} + \frac{k_{-a}}{k_{-t}} + 1} - k_{-t}\, e^{-\beta \Delta V} \right) A_0 \qquad \text{(A.7)}$$

In the limit of large transmembrane potentials (i.e., $k_{-t} \gg k_{-a}, k_{-l}$ and $e^{-\beta \Delta V} \to 0$), Eq. A7 reduces to

$$i_+ = (k_l + k_a) A_0 \qquad \text{(A.8)}$$

The result of Eq. A8 indicates that in the limit of large transmembrane potentials the amplitude of the outward current (inward chloride flux) becomes voltage-independent and is limited by chloride translocation to the vestibule via the lateral fenestrations and/or the apical pathway.

At negative voltage (outward chloride flux), chloride ions translocate through the transmembrane region first, enter the vestibule, and equilibrate with the extracellular milieu. Assuming that the rate constant for inward versus outward chloride translocation across the membrane decays exponentially with voltage, i.e., $k_{-t} = k_t \exp(-\beta \Delta V)$, Eq. A4 yields

$$[B]_{SS} = \frac{k_l + k_a + k_t}{k_{-l} + k_{-a} + k_t\, e^{-\beta \Delta V}} A_0 \qquad \text{(A.9)}$$

Assuming that k_{-l} and k_{-a} are voltage insensitive and that the electrostatic potential in the vestibule is the same as in the extracellular milieu, i.e., $k_{-l} = k_l$ and $k_{-a} = k_a$, in the limit of large transmembrane potentials, i.e., $e^{-\beta \Delta V} \to 0$, Eq. A9 reduces to

$$[B]_{SS} = \left(1 + \frac{k_t}{k_{-l} + k_{-a}} \right) A_0 \qquad \text{(A.10)}$$

which predicts that at negative voltage the vestibular concentration of chloride is higher than that in the extracellular milieu and it increases linearly with voltage (i.e., chloride ions are pumped into the vestibule). Assuming bidirectional chloride permeation across the membrane, i.e., $i_- = k_t A_0 - k_{-t}[B]_{SS}$, the net translocating current at negative voltage is

$$i_- = \left(1 - \frac{k_l + k_a + k_t}{k_{-l} + k_{-a} + k_t\, e^{-\beta \Delta V}}\, e^{-\beta \Delta V} \right) k_t A_0 \qquad \text{(A.11)}$$

which predicts that at negative voltage the net ionic current is not linear with voltage and its amplitude is sensitive to chloride translocation through the vestibule, i.e., k_l, k_a. However, in the limit of large transmembrane potentials (i.e., $e^{-\beta \Delta V} \to 0$), Eq. A11 yields

$$i_- = k_t A_0 \qquad \text{(A.12)}$$

consistent with Ohm's law.

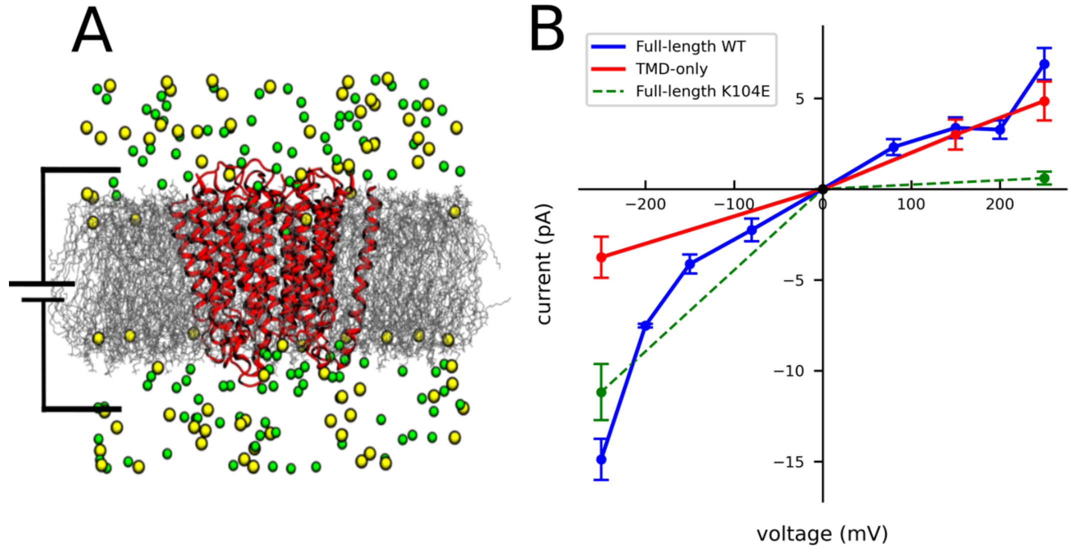

Fig. S1.

Related to Fig. 1. (**A**) Simulation setup for computational electrophysiology of the "TMD-only" GlyR model. To maintain an open-pore configuration, the simulations were carried out with harmonic restraints on the backbone as described in Ref. (15). (**B**) Calculated I-V curves from computational electrophysiology at various transmembrane potentials. Data-points for the full-length (cryo-EM construct) wild type GlyR-α1 (blue), TMD-only (red), and the full-length (cryo-EM construct) K104E GlyR mutant (green) are shown. Simulations were carried out in the presence of a 150 mM concentration of NaCl. For TMD-only Ionic currents were quantified from counting the number of ions permeating the pore per unit of time. Uncertainties were estimated assuming a Poisson distribution of the permeation events such that $\sigma = I/\sqrt{N}$ with I the current and N the number of permeation events (see **Table S1**).

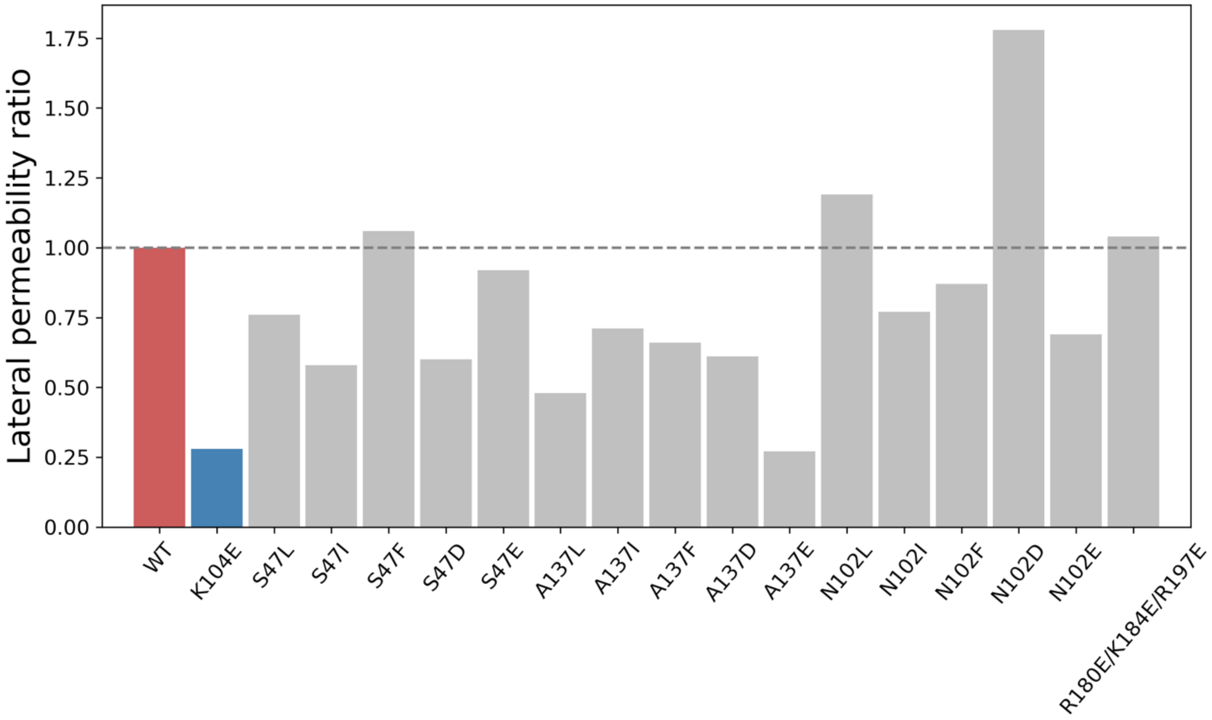

Fig. S2.

Related to Fig. 6. Results of the first *in-silico* screening campaign searching for mutations that reduce the lateral permeability of GlyR to chloride. In red is the wild-type protein. In blue is the literature ECD mutation known to strongly reduce the permeating current (22). In grey are mutations probed in this work *in silico*. The lateral permeability ratio corresponds to the ratio between the chloride permeation rate via the lateral tunnels over the chloride permeation rate though the transmembrane domain, which are both measured in computational electrophysiology at −280 mV and 150 mM concentration of NaCl (see *Methods*). This ratio quantifies the fraction of the chloride outward flux that uses the lateral pathways to exit the vestibule. A value < 1 indicates a reduction of chloride permeability via the lateral fenestrations relative to the wild type. In screening mode, the computational experiments were carried out for 200 ns with no replicates (n=1); see **Table S5**. The most promising mutations were then combined in double and triple mutants and analyzed in greater detail (Fig. 6 in *Main Text*).

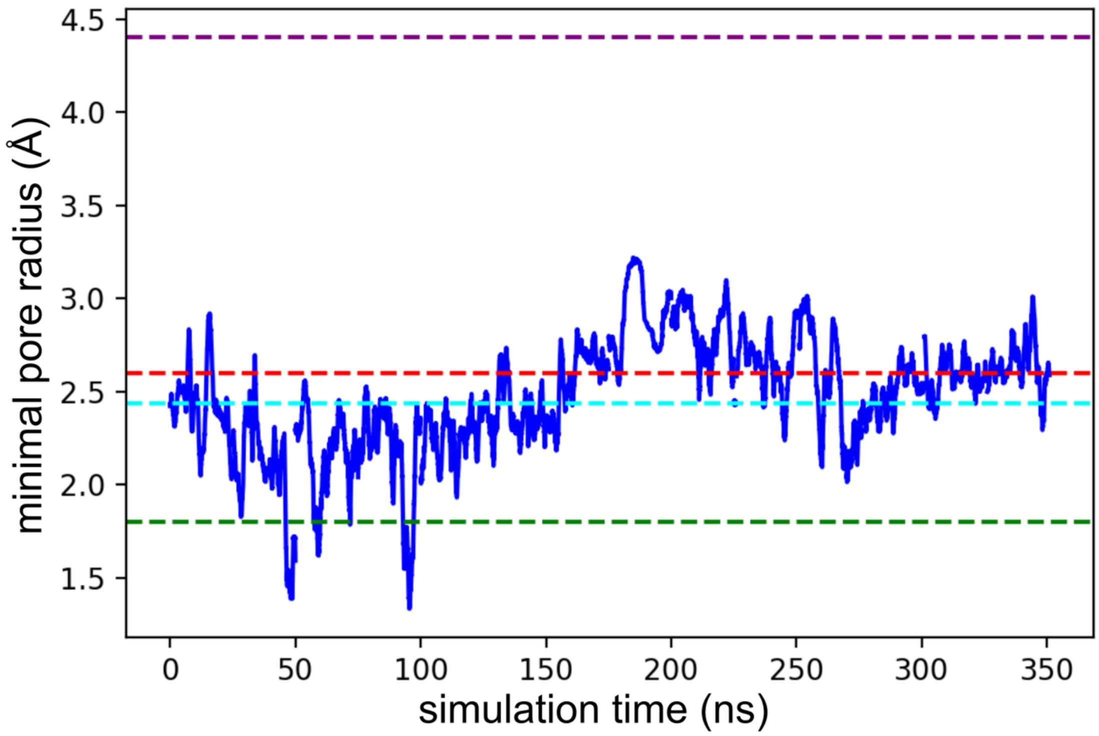

Fig. S3.

Related to *Materials and Methods*. Time series of the minimum pore radius during the equilibration of the MD-open model by unbiased molecular dynamics. Data-points in blue correspond to the minimum radius per simulation frame over the entire ion-transmembrane pore. The running average over 100 consecutive points is displayed. Cyan and purple dashed lines correspond to the minimum pore radius in the semi-open (PDB:3JAF) and the wide-open (PDB:3JAE) cryo-EM structures, respectively. The green dashed line corresponds to the radius of one chloride anion (68). The red dashed line indicates the minimum pore radius estimated by poly-atomic anions permeation experiments (5).

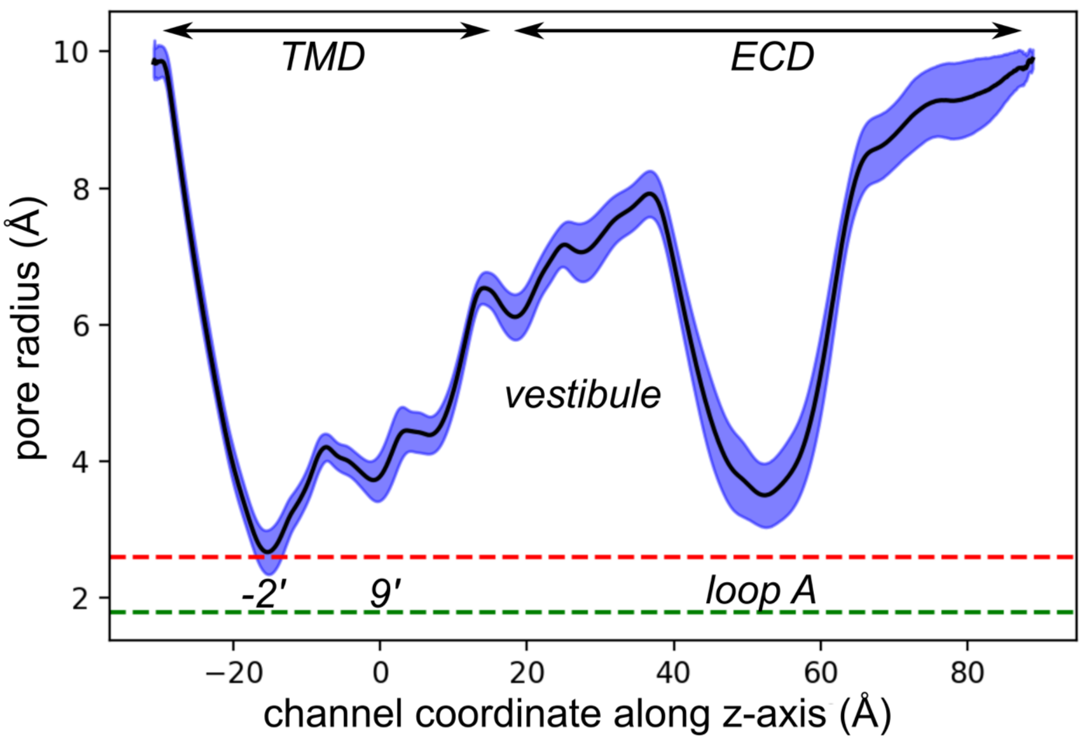

Fig. S4.

Related to *Materials and Methods*. Pore profile computed from the last 50 ns of the main 450 ns trajectory of the MD-open model. The thick black line corresponds to the mean pore radius over the 50 ns. The area filled in blue corresponds to the standard deviation. The red and green dashed lines represent the experimentally estimated minimum pore radius of GlyR-α1 (5) and the radius of a chloride anion (68), respectively.

```
                          -47----------59------105--137--180------197
GLYA1ARN      NSFGSIAETTMDYRV  ANEKGA  LAC  LRYCTKH  ARF
GLYA2ARN      NSFGSVTETTMDYRV  ANEKGA  LSC  LGYCTKH  VKF
GLYA3RN       NSFGSIAETTMDYRV  ANEKGA  LSC  LRYCTKH  VRF
GLYBRN        NSFGSIQETTMDYRV  ANEKSA  LSC  YGNCTKY  VIF
GABA1RN       TSFGPVSDHDMEYTI  HNGKKS  AEC  VDSGIVQ  THF
GABA2RN       TSFGPVSDTDMEYTI  HNGKKS  AEC  IGKETIK  AHF
GABA3RN       TSFGPVSDTDMEYTI  HNGKKS  AEC  VGTEIIR  THF
GABA4RN       TSFGPVSDVEMEYTM  RNGKKS  AEC  VSSETIK  VYF
GABA5RN       TSFGPVSDTEMEYTI  HNGKKS  AEC  VGTENIS  AHF
GABA6RN       TSFGPVSDVEMEYTM  RNGKKS  ADC  VSSETIK  VYF
GABB1RN       ASIDMVSEVNMDYTL  LNDKKS  AAC  MVSKKVE  LSF
GABB2RN       ASIDMVSEVNMDYTL  LNDKKS  AAC  LITKKVV  LSF
GABB3RN       ASIDMVSEVNMDYTL  LNDKKS  AAC  LVSRNVV  LSF
GABDRN        ASIDHISEANMEYTM  VNAKSA  VAC  FTTELMN  LHF
GABG1RN       NSIGPVDPINMEYTI  RNSRKS  AEC  NSTEISH  IFF
GABG2RN       NSIGPVNAINMEYTI  RNSKKA  AEC  NTTEVVK  VYF
GABG3RN       NSIGPVSSINMEYQI  RNSKTA  AEC  NTTEIVT  IYF
GABR2RN       ESLDSISEVDMDFTM  VHSKRS  AMC  TTSRLAF  INF
GABR3RN       ESIDSISEVNMDFTM  VHSKRS  AMC  ASSGLAF  INF
5-HT3MM       YAILNVDEKNQVLTT  NEFVD-  TAC  PQFKEFS  FYV
NICA7GG       MQIMDVDEKNQVLTT  YNSADE  SSC  GKRTESF  FTV
NICA1TC       IQLISVDEVNQIVET  YNNADG  SYC  GWKHWVY  YHF
NICDMM        SNLISLKEVEETLTT  ENNNDG  SSC  AKLNVDP  FYL
NICEMM        TNLISLNEKEETLTT  ENNIDG  STC  GMIRRYE  YTL
NICGMM        TNLISLNEREEALTT  ENNVDG  SSC  AKMLLDS  FYL
NICA2RN       AQLIDVDEKNQMMTT  YNNADG  SSC  GTYNSKK  YYF
NICA3RN       SQLVKVDEVNQIMET  YNNADG  SSC  GYKHEIK  YSL
NICA4RN       AQLIDVDEKNQMMTT  YNNADG  SSC  GTYNTRK  YAF
NICA5RN       SQLVDVDEKNQLMTT  FDNADG  SSC  GSKGNRT  YSF
NICA6RN       TQLANVDEVNQIMET  YNNAVG  SSC  GYKHDIK  YSF
NICA8GG       LQIIDVDEKNQVLIT  YNSADE  STC  GKRNELY  YTI
NICA9RR       SQIKDMDERNQILTA  YNKADD  SSC  AVKNVIS  FTL
NICB1MM       AQLISLNEKDEEMST  LNNNDG  SSC  SRLIQLP  FYL
NICB2RN       AQLISVHEREQIMTT  YNNADG  SAC  GRRNENP  YDF
NICB3RN       SQLVDVDEKNQLMTT  FENADG  SSC  GMKGNRR  YSF
NICB4RN       SQLISVNEREQIMTT  YNNADG  SAC  GRRTVNP  YDF
```

Fig. S5.

Related to *Discussion*. Sequence alignment of regions bordering the lateral fenestrations of a representative set of pLGICs. Taken from Ref. (38).

Table S1.

Related to Fig. 1 and Table 1. Computational electrophysiology results on the GlyR-α1 cryo-EM construct and the K104E mutant. The numerical results on the translocating current correspond to the number of chloride permeation events cumulated over multiple simulation runs at each voltage. Simulations were carried out in presence of a 150 mM symmetric concentration of NaCl. Ionic currents were quantified by counting the number of ions translocating the transmembrane pore over time. Error bars were estimated assuming a Poisson distribution of the permeation events, i.e., $\sigma = I/\sqrt{N}$ with I being the current and N the number of permeation events.

Voltage (mV)	−250	−200	−150	−80	80	150	200	250	−250 K104E	250 K104E
Cumulative simulation time (ns)	2045	1215	2520	926	2077	1663	2058	1442	1168	804
Number of replicas	10	10	6	4	10	6	6	10	6	6
Cumulative number of Apical permeations (Cl-)	9	6	6	5	1	0	1	3	15	0
Rate of Apical permeation (events/ns)	0.0044	0.0050	0.0024	0.0054	0.0005	0	0.0005	0.0021	0.0128	0
Cumulative number of Lateral permeations (Cl-)	173	70	110	65	95	60	86	73	22	2
Rate of Lateral permeation (events/ns)	0.085	0.058	0.044	0.070	0.046	0.036	0.042	0.050	0.019	0.003
Cumulative number of TMD permeations (Cl-)	173	55	62	13	28	35	42	61	79	3
Rate of TMD permeation (events/ns)	0.085	0.045	0.025	0.014	0.013	0.021	0.020	0.042	0.067	0.004
Current (pA)	−14.9 (±1.1)	−7.5 (±1.0)	−4.1 (±0.5)	−2.2 (±0.6)	2.3 (±0.4)	3.4 (±0.6)	3.3 (±0.5)	6.9 (±0.9)	−12.6 (±1.4)	0.6 (±0.3)
Conductance (pS)	59.5	37.6	27.6	28.1	28.9	22.5	16.3	27.6	50.5	2.4

Table S2.

Percentage of usage of the five lateral fenestrations in the ECD of GlyR from simulations carried on at ±250 mV.

Lateral tunnel	A	B	C	D	E
% of the ionic flux	28.2	1.2	8.5	61.9	0.3

Table S3.

Related to Fig. 3. Permeation rate through the lateral tunnels of various GlyR subtypes during simulations in the absence of an external transmembrane potential.

GlyR structure	GlyR α1 (MD-open)	GlyR α1 (PDB:6PM6)	GlyR α2β (PDB:5BKF)	GlyR α3 (PDB:5VDH)
Lateral permeation rate (permeation/ns)	0.04±0.01	0.07±0.02	0.06±0.04	0.07±0.01

Table S4.

Related to Fig. 6. Simulation of mutants GlyR, in the presence of a transmembrane potential of −280 mV and 150 mM of NaCl. For the mutants A137E and K104A/A137E, the number of replicas was increased to 3 and 6, respectively, because of too large statistical variability.

Mutation	D57I/ R59T	D57I	R59T	K104A/ G105D	K104A	G105D	A137E	K104/ A137E	K104A/ A137E	A137L/ N102L	A137I/ N102I	A137F / N102F	S47L/ N102L	S47I/ N102I	S47F/ N102F	S47L/ A137L	S47I/ A137I	S47F/ A137F	S47L/ N102L/ A137L	S47I / N102I / A137I	S47F / N102F/ A137F
Number of replicas × Simulation time (ns)	2× 100	2× 100	2× 100	2× 100	2× 100	2× 100	3× 100	2× 100	6× 100	2× 100	2× 100	2× 100	2× 100	2× 100	2× 100	2× 100	2× 100	2× 100	2× 100	2× 100	2× 100
Lateral permeability ratio	0.27 ±0.17	0.94 ±0.01	0.31 ±0.11	0.21 ±0.04	0.51 ±0.05	1.07 ±0.40	0.47 ±0.27	0.42 ±0.47	0.36 ±0.27	1.16 ±0.19	1.15 ±0.14	0.87 ±0.10	0.96 ±0.48	0.44 ±0.13	0.49 ±0.07	0.84 ±0.45	1.15 ±0.48	0.25 ±0.00	0.62 ±0.38	0.84 ±0.04	0.14 ±0.11
Cumulative number of lateral permeations	4	43	10	5	17	37	12	14	30	27	47	27	16	14	13	21	45	4	24	23	4
Cumulative number of TMD permeations	15	45	34	24	34	35	21	34	84	23	41	32	17	32	27	25	40	15	39	27	31

Table S5.

Related to Fig. S2. Simulations of mutants GlyR in the presence of a transmembrane potential of −280 mV and 150 mM of NaCl. All experiments with single-point mutants were carried out without replicates so that error bars on the lateral permeability ratio could not be quantified.

Mutation	S63L	S63I	S63F	S63D	S63E	A153L	A153I	A153F	A153D	A153E	N118L	N118I	N118F	N118D	N118E	R180E/ K184E/ R197E
Number of replicas × Simulation time (ns)	1× 200	1× 200	1× 200	1× 200	1× 200	1× 200	1× 200	1× 200	1× 200	1× 200	1× 200	1× 200	1× 200	1× 200	1× 200	1× 200
Lateral permeability ratio	0.8	0.6	1.1	0.6	0.9	0.5	0.7	0.7	0.6	0.3	1.2	0.8	0.9	1.8	0.7	1.0
Cumulative number of lateral permeations (Cl^-)	14	14	19	12	46	44	16	16	17	3	33	46	10	13	7	25
Cumulative number of transmembrane permeations (Cl^-)	18	24	18	19	49	93	23	24	28	12	28	61	11	7	11	24

Table S6.

Related to Fig. 7. EC50 values and nHill calculated for WT and mutants obtained by establishment of dose-response curves following recordings via TEVC on Xenopus oocytes.

**Receptor type**	**EC_{50}**	**n_{Hill}**	**n**
Wild-type	158.1 ± 11.69	1.905 ± 0.217	6
K104E	136.4 ± 9.207	2.096 ± 0.253	8
K104A/A137E	103.2 ± 21.02	0.9142 ± 0.124	6
S47F/A137F	320.3 ± 70.73	1.181 ± 0.209	7

Table S7.

Related to Fig. 7. Mean current amplitudes determined for the three mutated constructs and the wild-type GlyR by outside-out single-channel recordings on transiently transfected HEK293 cells upon *4s* application of 1-10 µM glycine.

**Wild type**				**K104E**			**K104A/A137E**			**S47F/A137F**		
	n	**Mean current (pA)**	**SD**	***n***	**Mean current (pA)**	**SD**	***n***	**Mean current (pA)**	**SD**	***n***	**Mean current (pA)**	**SD**
**-100**	5	-8.378	0.898	6	-6.363	1.074	5	-4.957	0.814	6	-7.424	0.466
**-80**	5	-6.556	0.413	7	-4.857	1.028	5	-4.295	0.939	6	-5.851	0.344
**-60**	5	-4.580	0.471	7	-3.729	0.608	5	-2.869	0.922	6	-4.082	0.233
**-40**	5	-2.935	0.238	7	-1.850	0.559	5	-1.556	0.601	6	-2.670	0.165
**-20**	5	-1.198	0.282	7	-0.932	0.303	5	-0.653	0.226	6	-1.237	0.231
**0**	5	0	0	7	0	0	5	0	0	6	0	0
**20**	5	1.460	0.085	7	0.686	0.181	5	0.604	0.342	6	0.777	0.121
**40**	5	2.644	0.344	7	1.080	0.151	5	1.040	0.298	6	1.482	0.185
**60**	5	3.989	0.561	5	1.425	0.100	5	1.187	0.281	6	1.980	0.316
**80**	5	4.798	0.336	6	1.693	0.086	5	1.468	0.157	6	2.535	0.357
**100**	4	6.191	0.488	2	2.069	0.071	4	1.590	0.147	5	2.801	0.408

Table S8.

Related to *Discussion*. Analysis of lateral fenestrations in the active/desensitized structures of various pLGICs. A selection of recent X-ray and cryo-EM structures of pLGICs was analyzed using the webserver MOLEonline (https://mole.upol.cz). From a structural point of view, lateral fenestrations or tunnels can be detected in all pLGICs but their shape is heterogeneous and varies among the subtypes or after relaxation by Molecular Dynamics (MD-open). From a functional perspective, the lateral fenestrations in MD-open, 6PM6, 5BKF, and 5VDH structures were shown to be ion permeable *in silico* (see *Main Text*). The reported tunnel radius corresponds to the radius at the constriction point.

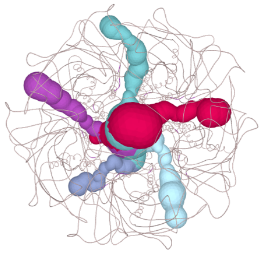<br>Receptor: GlyR α1 (Active)<br>PDB: MD snapshot<br>Tunnels radius:1.4-2.2Å	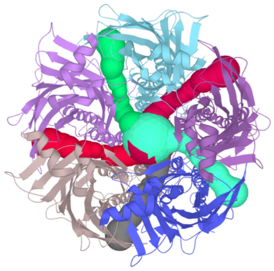<br>Receptor: GlyR α1 (wide-open)<br>PDB: 3JAE<br>Tunnels radius: 2Å	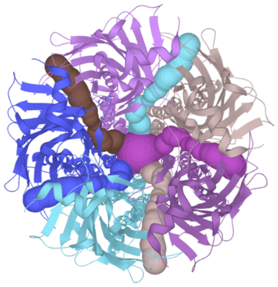<br>Receptor: GlyR α1 (Active)<br>PDB: 6PM6<br>Tunnels radius: 2Å	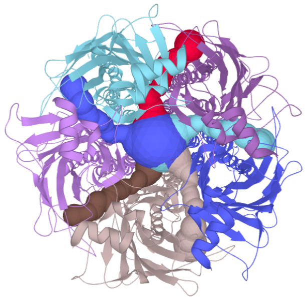<br>Receptor: GlyR α2β (Desensitized)<br>PDB: 5BKF<br>Tunnels radius: 2.3Å
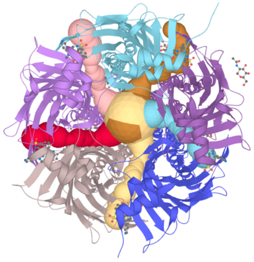<br>Receptor: GlyR α3 (Desensitized)<br>PDB: 5VDH<br>Tunnels radius: 2.0-2.1Å	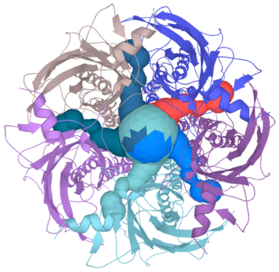<br>Receptor: $GABA_AR$ β3 (Desensitized)<br>PDB: 7A5V<br>Tunnels radius: 1.7Å	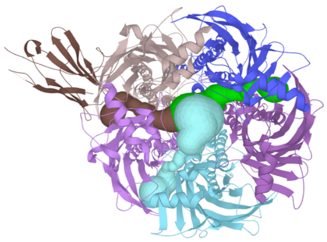<br>Receptor: $GABA_AR$ α1β3γ2 (Desensitized)<br>PDB: 7HUP<br>Tunnels radius: 1.8-1.9Å	
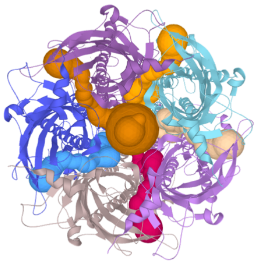<br>Receptor: $5\text{-}HT_3R$ (Active state 2)<br>PDB: 6DG8<br>Tunnels radius: 1.7-2.1 Å	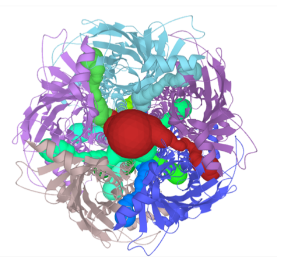<br>Receptor: nAchR α7 (Active)<br>PDB: 7KOX<br>Tunnels radius ECD 1.3Å<br>Tunnels radius ICD 1.4-1.7Å	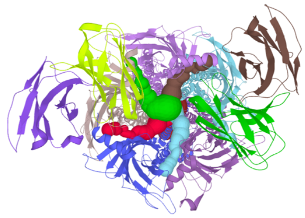<br>Receptor: nAchR α3β4 (Desensitized)<br>PDB: 6PV7<br>Tunnels radius ECD: 1.5-2Å<br>Tunnels radius ICD: 1.8 Å	

REFERENCES AND NOTES

1. J. W. Lynch, Molecular structure and function of the glycine receptor chloride channel. *Physiol. Rev.* **84**, 1051–1095 (2004).

2. S. Dutertre, C.-M. Becker, H. Betz, Inhibitory glycine receptors: An update. *J. Biol. Chem.* **287**, 40216–40223 (2012).

3. A. Bode, J. W. Lynch, The impact of human hyperekplexia mutations on glycine receptor structure and function. *Mol. Brain* **7**, 2 (2014).

4. J. Yu, H. Zhu, R. Lape, T. Greiner, J. Du, W. Lü, L. Sivilotti, E. Gouaux, Mechanism of gating and partial agonist action in the glycine receptor. *Cell* **184**, 957–968.e21 (2021).

5. J. Bormann, O. P. Hamill, B. Sakmann, Mechanism of anion permeation through channels gated by glycine and gamma-aminobutyric acid in mouse cultured spinal neurones. *J. Physiol.* **385**, 243–286 (1987).

6. A. Keramidas, A. J. Moorhouse, K. D. Pierce, P. R. Schofield, P. H. Barry, Cation-selective mutations in the M2 domain of the inhibitory glycine receptor channel reveal determinants of ion-charge selectivity. *J. Gen. Physiol.* **119**, 393–410 (2002).

7. A. Keramidas, A. J. Moorhouse, C. R. French, P. R. Schofield, P. H. Barry, M2 pore mutations convert the glycine receptor channel from being anion- to cation-selective. *Biophys. J.* **79**, 247–259 (2000).

8. G. Duret, C. Van Renterghem, Y. Weng, M. Prevost, G. Moraga-Cid, C. Huon, J. M. Sonner, P.-J. Corringer, Functional prokaryotic–eukaryotic chimera from the pentameric ligand-gated ion channel family. *Proc. Natl. Acad. Sci. U.S.A.* **108**, 12143–12148 (2011).

9. J. Bormann, N. Rundström, H. Betz, D. Langosch, Residues within transmembrane segment M2 determine chloride conductance of glycine receptor homo- and hetero-oligomers. *EMBO J.* **12**, 3729–3737 (1993).

10. J. Du, W. Lü, S. Wu, Y. Cheng, E. Gouaux, Glycine receptor mechanism elucidated by electron cryo-microscopy. *Nature* **526**, 224–229 (2015).

11. X. Huang, P. L. Shaffer, S. Ayube, H. Bregman, H. Chen, S. G. Lehto, J. A. Luther, D. J. Matson, S. I. McDonough, K. Michelsen, M. H. Plant, S. Schneider, J. R. Simard, Y. Teffera, S. Yi, M. Zhang, E. F. DiMauro, J. Gingras, Crystal structures of human glycine receptor α3 bound to a novel class of analgesic potentiators. *Nat. Struct. Mol. Biol.* **24**, 108–113 (2017).

12. X. Huang, H. Chen, P. L. Shaffer, Crystal structures of human GlyRα3 bound to Ivermectin. *Structure* **25**, 945–950.e2 (2017).

13. X. Huang, H. Chen, K. Michelsen, S. Schneider, P. L. Shaffer, Crystal structure of human glycine receptor-α3 bound to antagonist strychnine. *Nature* **526**, 277–280 (2015).

14. A. Kumar, S. Basak, S. Rao, Y. Gicheru, M. L. Mayer, M. S. P. Sansom, S. Chakrapani, Mechanisms of activation and desensitization of full-length glycine receptor in lipid nanodiscs. *Nat. Commun.* **11**, 3752 (2020).

15. A. H. Cerdan, N. É. Martin, M. Cecchini, An ion-permeable state of the glycine receptor captured by molecular dynamics. *Structure* **26**, 1555–1562.e4 (2018).

16. A. H. Cerdan, M. Cecchini, On the functional annotation of open-channel structures in the glycine receptor. *Structure* **28**, 690–693.e3 (2020).

17. M. Moroni, I. Biro, M. Giugliano, R. Vijayan, P. C. Biggin, M. Beato, L. G. Sivilotti, Chloride ions in the pore of glycine and GABA channels shape the time course and voltage dependence of agonist currents. *J. Neurosci.* **31**, 14095–14106 (2011).

18. C. O. Lara, C. F. Burgos, T. Silva-Grecchi, C. Muñoz-Montesino, L. G. Aguayo, J. Fuentealba, P. A. Castro, J. L. Guzmán, P.-J. Corringer, G. E. Yévenes, G. Moraga-Cid, Large intracellular domain-dependent effects of positive allosteric modulators on glycine receptors. *ACS Chem. Nerosci.* **10**, 2551–2559 (2019).

19. J. Ivica, R. Lape, V. Jazbec, J. Yu, H. Zhu, E. Gouaux, M. G. Gold, L. G. Sivilotti, The intracellular domain of homomeric glycine receptors modulates agonist efficacy. *J. Biol. Chem.* **296**, 100387 (2021).

20. J. E. Carland, M. A. Cooper, S. Sugiharto, H.-J. Jeong, T. M. Lewis, P. H. Barry, J. A. Peters, J. J. Lambert, A. J. Moorhouse, Characterization of the effects of charged residues in the intracellular loop on ion permeation in α1 glycine receptor channels. *J. Biol. Chem.* **284**, 2023–2030 (2009).

21. M. Brams, E. A. Gay, J. C. Sáez, A. Guskov, R. van Elk, R. C. van der Schors, S. Peigneur, J. Tytgat, S. V. Strelkov, A. B. Smit, J. L. Yakel, C. Ulens, Crystal structures of a cysteine-modified mutant in loop D of acetylcholine-binding protein. *J. Biol. Chem.* **286**, 4420–4428 (2011).

22. M. Moroni, J. O. Meyer, C. Lahmann, L. G. Sivilotti, In glycine and $GABA_A$ channels, different subunits contribute asymmetrically to channel conductance via residues in the extracellular domain. *J. Biol. Chem.* **286**, 13414–13422 (2011).

23. S. B. Hansen, H.-L. Wang, P. Taylor, S. M. Sine, An ion selectivity filter in the extracellular domain of Cys-loop receptors reveals determinants for ion conductance. *J. Biol. Chem.* **283**, 36066–36070 (2008).

24. A. J. Moorhouse, A. Keramidas, A. Zaykin, P. R. Schofield, P. H. Barry, Single channel analysis of conductance and rectification in cation-selective, mutant glycine receptor channels. *J. Gen. Physiol.* **119**, 411–425 (2002).

25. S. Scott, J. W. Lynch, A. Keramidas, Correlating structural and energetic changes in glycine receptor activation. *J. Biol. Chem.* **290**, 5621–5634 (2015).

26. L. Pravda, D. Sehnal, D. Toušek, V. Navrátilová, V. Bazgier, K. Berka, R. Svobodová Vařeková, J. Koča, M. Otyepka, MOLEonline: A web-based tool for analyzing channels, tunnels and pores (2018 update). *Nucleic Acids Res.* **46**, W368–W373 (2018).

27. B. Hille, *Ion Channels of Excitable Membranes* (Oxford Univ. Press, ed. 3, 2018).

28. P. Aryal, M. S. P. Sansom, S. J. Tucker, Hydrophobic gating in ion channels. *J. Mol. Biol.* **427**, 121–130 (2015).

29. J. L. Galzi, S. J. Edelstein, J. Changeux, The multiple phenotypes of allosteric receptor mutants. *Proc. Natl. Acad. Sci. U.S.A.* **93**, 1853–1858 (1996).

30. R. J. Howard, Elephants in the dark: Insights and incongruities in pentameric ligand-gated ion channel models. *J. Mol. Biol.* **433**, 167128 (2021).

31. Á. Nemecz, M. S. Prevost, A. Menny, P.-J. Corringer, Emerging molecular mechanisms of signal transduction in pentameric ligand-gated ion channels. *Neuron* **90**, 452–470 (2016).

32. C. M. Noviello, A. Gharpure, N. Mukhtasimova, R. Cabuco, L. Baxter, D. Borek, S. M. Sine, R. E. Hibbs, Structure and gating mechanism of the α7 nicotinic acetylcholine receptor. *Cell* **184**, 2121–2134.e13 (2021).

33. Z. Lu, Mechanism of rectification in inward-rectifier K+ channels. *Annu. Rev. Physiol.* **66**, 103–129 (2004).

34. D. Bowie, M. L. Mayer, Inward rectification of both AMPA and kainate subtype glutamate receptors generated by polyamine-mediated ion channel block. *Neuron* **15**, 453–462 (1995).

35. C. M. Armstrong, S. Hollingworth, A perspective on Na and K channel inactivation. *J. Gen. Physiol.* **150**, 7–18 (2018).

36. A. P. Haghighi, E. Cooper, A molecular link between inward rectification and calcium permeability of neuronal nicotinic acetylcholine α3β4 and α4β2 receptors. *J. Neurosci.* **20**, 529–541 (2000).

37. X. Xiu, N. L. Puskar, J. A. P. Shanata, H. A. Lester, D. A. Dougherty, Nicotine binding to brain receptors requires a strong cation–π interaction. *Nature* **458**, 534–537 (2009).

38. N. Le Novère, P.-J. Corringer, J.-P. Changeux, Improved secondary structure predictions for a nicotinic receptor subunit: Incorporation of solvent accessibility and experimental data into a two-dimensional representation. *Biophys. J.* **76**, 2329–2345 (1999).

39. G. D. Cymes, C. Grosman, Identifying the elusive link between amino acid sequence and charge selectivity in pentameric ligand-gated ion channels. *Proc. Natl. Acad. Sci. U.S.A.* **113**, E7106–E7115 (2016).

40. P. S. Miller, A. R. Aricescu, Crystal structure of a human $GABA_A$ receptor. *Nature* **512**, 270–275 (2014).

41. S. Zhu, C. M. Noviello, J. Teng, R. M. Walsh, J. J. Kim, R. E. Hibbs, Structure of a human synaptic $GABA_A$ receptor. *Nature* **559**, 67–72 (2018).

42. D. Di Maio, B. Chandramouli, G. Brancato, Pathways and barriers for ion translocation through the 5-HT3A receptor channel. *PLOS ONE.* **10**, e0140258 (2015).

43. H. Hu, Á. Nemecz, C. Van Renterghem, Z. Fourati, L. Sauguet, P.-J. Corringer, M. Delarue, Crystal structures of a pentameric ion channel gated by alkaline pH show a widely open pore and identify a cavity for modulation. *Proc. Natl. Acad. Sci. U.S.A.* **115**, E3959–E3968 (2018).

44. M. Brams, C. Govaerts, K. Kambara, K. L. Price, R. Spurny, A. Gharpure, E. Pardon, G. L. Evans, D. Bertrand, S. C. Lummis, R. E. Hibbs, J. Steyaert, C. Ulens, Modulation of the *Erwinia* ligand-gated ion channel (ELIC) and the $5\text{-}HT_3$ receptor via a common vestibule site. *eLife* **9**, e51511 (2020).

45. H. Yu, X.-C. Bai, W. Wang, Characterization of the subunit composition and structure of adult human glycine receptors. *Neuron* **109**, 2707–2716.e6 (2021).

46. S. Phulera, H. Zhu, J. Yu, D. P. Claxton, N. Yoder, C. Yoshioka, E. Gouaux, Cryo-EM structure of the benzodiazepine-sensitive α1β1γ2S tri-heteromeric $GABA_A$ receptor in complex with GABA. *eLife* **7**, e39383 (2018).

47. D. Laverty, R. Desai, T. Uchański, S. Masiulis, W. J. Stec, T. Malinauskas, J. Zivanov, E. Pardon, J. Steyaert, K. W. Miller, A. R. Aricescu, Cryo-EM structure of the human α1β3γ2 $GABA_A$ receptor in a lipid bilayer. *Nature* **565**, 516–520 (2019).

48. T. Kawate, J. L. Robertson, M. Li, S. D. Silberberg, K. J. Swartz, Ion access pathway to the transmembrane pore in P2X receptor channels. *J. Gen. Physiol.* **137**, 579–590 (2011).

49. D. S. K. Samways, B. S. Khakh, S. Dutertre, T. M. Egan, Preferential use of unobstructed lateral portals as the access route to the pore of human ATP-gated ion channels (P2X receptors). *Proc. Natl. Acad. Sci. U.S.A.* **108**, 13800–13805 (2011).

50. O. S. Smart, J. G. Neduvelil, X. Wang, B. A. Wallace, M. S. P. Sansom, HOLE: A program for the analysis of the pore dimensions of ion channel structural models. *J. Mol. Graph.* **14**, 354–360 (1996).

51. S. Jo, T. Kim, V. G. Iyer, W. Im, CHARMM-GUI: A web-based graphical user interface for CHARMM. *J. Comput. Chem.* **29**, 1859–1865 (2008).

52. E. L. Wu, X. Cheng, S. Jo, H. Rui, K. C. Song, E. M. Dávila-Contreras, Y. Qi, J. Lee, V. Monje-Galvan, R. M. Venable, J. B. Klauda, W. Im, CHARMM-GUI *Membrane Builder* toward realistic biological membrane simulations. *J. Comput. Chem.* **35**, 1997–2004 (2014).

53. B. R. Brooks, C. L. Brooks III, A. D. Mackerell Jr., L. Nilsson, R. J. Petrella, B. Roux, Y. Won, G. Archontis, C. Bartels, S. Boresch, A. Caflisch, L. Caves, Q. Cui, A. R. Dinner, M. Feig, S. Fischer, J. Gao, M. Hodoscek, W. Im, K. Kuczera, T. Lazaridis, J. Ma, V. Ovchinnikov, E. Paci, R. W. Pastor, C. B. Post, J. Z. Pu, M. Schaefer, B. Tidor, R. M. Venable, H. L. Woodcock, X. Wu, W. Yang, D. M. York, M. Karplus, CHARMM: The biomolecular simulation program. *J. Comput. Chem.* **30**, 1545–1614 (2009).

54. M. J. Abraham, T. Murtola, R. Schulz, S. Páll, J. C. Smith, B. Hess, E. Lindahl, GROMACS: High performance molecular simulations through multi-level parallelism from laptops to supercomputers. *SoftwareX* **1-2**, 19–25 (2015).

55. R. B. Best, X. Zhu, J. Shim, P. E. M. Lopes, J. Mittal, M. Feig, A. D. MacKerell Jr., Optimization of the additive CHARMM all-atom protein force field targeting improved sampling of the backbone ϕ, ψ and side-chain χ_1 and χ_2 dihedral angles. *J. Chem. Theory Comput.* **8**, 3257–3273 (2012).

56. J. B. Klauda, R. M. Venable, J. A. Freites, J. W. O'Connor, D. J. Tobias, C. Mondragon-Ramirez, I. Vorobyov, A. D. MacKerell Jr., R. W. Pastor, Update of the CHARMM all-atom additive force field for lipids: Validation on six lipid types. *J. Phys. Chem. B* **114**, 7830–7843 (2010).

57. J. Huang, S. Rauscher, G. Nawrocki, T. Ran, M. Feig, B. L. de Groot, H. Grubmüller, A. D. MacKerell Jr., CHARMM36m: An improved force field for folded and intrinsically disordered proteins. *Nat. Methods* **14**, 71–73 (2017).

58. J. Lee, X. Cheng, J. M. Swails, M. S. Yeom, P. K. Eastman, J. A. Lemkul, S. Wei, J. Buckner, J. C. Jeong, Y. Qi, S. Jo, V. S. Pande, D. A. Case, C. L. Brooks, A. D. MacKerell, J. B. Klauda, W. Im, CHARMM-GUI input generator for NAMD, GROMACS, AMBER, OpenMM, and CHARMM/OpenMM simulations using the CHARMM36 additive force field. *J. Chem. Theory Comput.* **12**, 405–413 (2016).

59. G. Bussi, D. Donadio, M. Parrinello, Canonical sampling through velocity rescaling. *J. Chem. Phys.* **126**, 014101 (2007).

60. M. Parrinello, A. Rahman, Polymorphic transitions in single crystals: A new molecular dynamics method. *J. Appl. Phys.* **52**, 7182–7190 (1981).

61. B. Hess, H. Bekker, H. J. C. Berendsen, J. G. E. M. Fraaije, LINCS: A linear constraint solver for molecular simulations. *J. Comput. Chem.* **18**, 1463–1472 (1997).

62. T. Darden, D. York, L. Pedersen, Particle mesh Ewald: An $N \cdot \log(N)$ method for Ewald sums in large systems. *J. Chem. Phys.* **98**, 10089–10092 (1993).

63. B. Roux, The membrane potential and its representation by a constant electric field in computer simulations. *Biophys. J.* **95**, 4205–4216 (2008).

64. M. Seeber, M. Cecchini, F. Rao, G. Settanni, A. Caflisch, Wordom: A program for efficient analysis of molecular dynamics simulations. *Bioinformatics* **23**, 2625–2627 (2007).

65. M. Seeber, A. Felline, F. Raimondi, S. Muff, R. Friedman, F. Rao, A. Caflisch, F. Fanelli, Wordom: A user-friendly program for the analysis of molecular structures, trajectories, and free energy surfaces. *J. Comput. Chem.* **32**, 1183–1194 (2011).

66. M. Sotomayor, V. Vásquez, E. Perozo, K. Schulten, Ion conduction through MscS as determined by electrophysiology and simulation. *Biophys. J.* **92**, 886–902 (2007).

67. W. Humphrey, A. Dalke, K. Schulten, VMD: Visual molecular dynamics. *J. Mol. Graph.* **14**, 33–38 (1996).

68. E. R. Nightingale Jr., Phenomenological theory of ion solvation. Effective radii of hydrated ions. *J. Phys. Chem.* **63**, 1381–1387 (1959).
